# Absolute risk and predictors of the growth of acute spontaneous intracerebral haemorrhage: a systematic review and meta-analysis of individual patient data

**DOI:** 10.1016/S1474-4422(18)30253-9

**Published:** 2018-10

**Authors:** Rustam Al-Shahi Salman, Joseph Frantzias, Robert J Lee, Patrick D Lyden, Thomas W K Battey, Alison M Ayres, Joshua N Goldstein, Stephan A Mayer, Thorsten Steiner, Xia Wang, Hisatomi Arima, Hitoshi Hasegawa, Makoto Oishi, Daniel A Godoy, Luca Masotti, Dar Dowlatshahi, David Rodriguez-Luna, Carlos A Molina, Dong-Kyu Jang, Antonio Davalos, José Castillo, Xiaoying Yao, Jan Claassen, Bastian Volbers, Seiji Kazui, Yasushi Okada, Shigeru Fujimoto, Kazunori Toyoda, Qi Li, Jane Khoury, Pilar Delgado, José Álvarez Sabín, Mar Hernández-Guillamon, Luis Prats-Sánchez, Chunyan Cai, Mahesh P Kate, Rebecca McCourt, Chitra Venkatasubramanian, Michael N Diringer, Yukio Ikeda, Hans Worthmann, Wendy C Ziai, Christopher D d'Esterre, Richard I Aviv, Peter Raab, Yasuo Murai, Allyson R Zazulia, Kenneth S Butcher, Seyed Mohammad Seyedsaadat, James C Grotta, Joan Martí-Fàbregas, Joan Montaner, Joseph Broderick, Haruko Yamamoto, Dimitre Staykov, E Sander Connolly, Magdy Selim, Rogelio Leira, Byung Hoo Moon, Andrew M Demchuk, Mario Di Napoli, Yukihiko Fujii, Craig S Anderson, Jonathan Rosand, VISTA-ICH Collaboration, Daniel F Hanley, Kenneth S Butcher, Stephen Davis, Barbara Gregson, Kennedy R Lees, Patrick D Lyden, Stephan A Mayer, Keith W Muir, Thorsten Steiner, ICH Growth Individual Patient Data Meta-analysis Collaborators, Peng Xie, Babak Bakhshayesh, Mark McDonald, Thomas Brott, Paolo Pennati, Adrian R Parry-Jones, Craig J Smith, Stephen J Hopkins, Mark Slevin, Veronica Campi, Puneetpal Singh, Francesca Papa, Aurel Popa-Wagner, Valeria Tudorica, Ryo Takagi, Akira Teramoto, Karin Weissenborn, Heinrich Lanfermann

**Affiliations:** a Centre for Clinical Brain Sciences, University of Edinburgh, Edinburgh, UK; b Usher Institute of Population Health Sciences and Informatics, University of Edinburgh, Edinburgh, UK; c Department of Neurosurgery, King's College Hospital, London, UK; d Department of Neurology, Cedars-Sinai Medical Center, Los Angeles, California, USA; e Department of Neurology, Massachusetts General Hospital, Boston, MA, USA; f Department of Emergency Medicine, Massachusetts General Hospital, Boston, MA, USA; g Department of Neurology, Henry Ford Health System, Detroit, MI, USA; h Department of Neurology, Klinikum Frankfurt Höchst, Frankfurt, Germany; i Department of Neurology, Heidelberg University Hospital, Heidelberg, Germany; j The George Institute for Global Health, Faculty of Medicine, University of New South Wales, Sydney, NSW, Australia; k The George Institute for Global Health, China at Peking University Health Science Center, Beijing, China; l Department of Neurosurgery, Brain Research Institute, Niigata University, Niigata, Japan; m Intensive Care Unit, Hospital Interzonal de Agudos “San Juan Bautista”, Catamarca, Argentina; n Internal Medicine, Santa Maria Nuova Hospital, Florence, Italy; o Department of Medicine (Neurology), University of Ottawa and Ottawa Hospital Research Institute, Ottawa, ON, Canada; p Stroke Unit, Department of Neurology, Vall d'Hebron University Hospital, Vall d'Hebron Research Institute, Autonomous University of Barcelona, Barcelona, Spain; q Department of Neurosciences, Hospital Germans Trias i Pujol, Autonomous University of Barcelona, Barcelona, Spain; r Department of Neurosurgery, Incheon St Mary's Hospital, College of Medicine, The Catholic University of Korea, Incheon, Korea; s Department of Neurology, University Clinical Hospital of Santiago, Santiago de Compostela, Spain; t Department of Neurology, Ren Ji Hospital, School of Medicine, Shanghai Jiao Tong University, Shanghai, China; u Department of Neurology, Columbia University, College of Physicians and Surgeons, New York, NY, USA; v Department of Neurosurgery, Columbia University, College of Physicians and Surgeons, New York, NY, USA; w Department of Neurology, Inselspital, Bern University Hospital, University of Bern, Switzerland; x Kazui Medical Office, Ikeda, Osaka, Japan; y Department of Cerebrovascular Medicine and Neurology, National Hospital Organisation Kyushu Medical Centre, Fukuoka, Japan; z Division of Neurology, Department of Medicine, Jichi Medical University, Shimotsuke, Tochigi, Japan; aa Department of Cerebrovascular Medicine, National Cerebral and Cardiovascular Center, Suita, Osaka, Japan; ab Department of Neurology, ThE First Affiliated Hospital of Chongqing Medical University, Chongqing, China; ac University of Cincinnati Medical Centre, Cincinnati, OH, USA; ad Neurovascular Research Lab, Neurovascular Section, Institut de Recerca and Hospital Vall d'Hebron, Barcelona, Spain; ae Department of Neurology, Hospital de la Santa Creu i Sant Pau, IIB Sant Pau, Barcelona, Spain; af Centre for Clinical and Translational Sciences, McGovern Medical School at the University of Texas Health Science Center, Houston, TX, USA; ag Department of Neurology, University of Alberta, Edmonton, AB, Canada; ah Division of Stroke and Neurocritical Care, Stanford University, Palo Alto, CA, USA; ai Department of Neurology and Neurological Surgery, Washington University, St Louis, MO, USA; aj Department of Neurosurgery, Tokyo Medical University Hachioji Medical Center, Tokyo, Japan; ak Department of Neurology, Hannover Medical School, Hannover, Germany; al Institute of Diagnostic and Interventional Neuroradiology, Hannover Medical School, Hannover, Germany; am Johns Hopkins University School of Medicine, Department of Neurology, Anaesthesia and Critical Care Medicine, Division of Neurocritical Care, Baltimore, MD, USA; an Department of Radiology, Foothills Medical Centre, University of Calgary, Calgary, AB, Canada; ao Department of Medical Imaging, Sunnybrook Health Sciences Centre, University of Toronto, Toronto, ON, Canada; ap Department of Neurological Surgery, Nippon Medical School, Tokyo, Japan; aq Department of Radiology, Mayo Clinic, Rochester, MN, USA; ar Memorial Hermann Hospital, Clinical Innovation and Research Institute, Houston, TX, USA; as Center for Advanced Clinical and Translational Sciences, National Cerebral and Cerebrovascular Center, Suita, Osaka, Japan; at Department of Neurology, Hospital of the Brothers of St John, Eisenstadt, Austria; au Department of Neurology, Beth Israel Deaconess Medical Center, Boston, MA, USA; av Department of Clinical Neurosciences and Department of Radiology, Hotchkiss Brain Institute, Cumming School of Medicine, University of Calgary, Calgary, AB, Canada; aw Neurological Service, San Camillo de'Lellis General Hospital, Rieti, Italy

## Abstract

**Background:**

Intracerebral haemorrhage growth is associated with poor clinical outcome and is a therapeutic target for improving outcome. We aimed to determine the absolute risk and predictors of intracerebral haemorrhage growth, develop and validate prediction models, and evaluate the added value of CT angiography.

**Methods:**

In a systematic review of OVID MEDLINE—with additional hand-searching of relevant studies' bibliographies— from Jan 1, 1970, to Dec 31, 2015, we identified observational cohorts and randomised trials with repeat scanning protocols that included at least ten patients with acute intracerebral haemorrhage. We sought individual patient-level data from corresponding authors for patients aged 18 years or older with data available from brain imaging initially done 0·5–24 h and repeated fewer than 6 days after symptom onset, who had baseline intracerebral haemorrhage volume of less than 150 mL, and did not undergo acute treatment that might reduce intracerebral haemorrhage volume. We estimated the absolute risk and predictors of the primary outcome of intracerebral haemorrhage growth (defined as >6 mL increase in intracerebral haemorrhage volume on repeat imaging) using multivariable logistic regression models in development and validation cohorts in four subgroups of patients, using a hierarchical approach: patients not taking anticoagulant therapy at intracerebral haemorrhage onset (who constituted the largest subgroup), patients taking anticoagulant therapy at intracerebral haemorrhage onset, patients from cohorts that included at least some patients taking anticoagulant therapy at intracerebral haemorrhage onset, and patients for whom both information about anticoagulant therapy at intracerebral haemorrhage onset and spot sign on acute CT angiography were known.

**Findings:**

Of 4191 studies identified, 77 were eligible for inclusion. Overall, 36 (47%) cohorts provided data on 5435 eligible patients. 5076 of these patients were not taking anticoagulant therapy at symptom onset (median age 67 years, IQR 56–76), of whom 1009 (20%) had intracerebral haemorrhage growth. Multivariable models of patients with data on antiplatelet therapy use, data on anticoagulant therapy use, and assessment of CT angiography spot sign at symptom onset showed that time from symptom onset to baseline imaging (odds ratio 0·50, 95% CI 0·36–0·70; p<0·0001), intracerebral haemorrhage volume on baseline imaging (7·18, 4·46–11·60; p<0·0001), antiplatelet use (1·68, 1·06–2·66; p=0·026), and anticoagulant use (3·48, 1·96–6·16; p<0·0001) were independent predictors of intracerebral haemorrhage growth (C-index 0·78, 95% CI 0·75–0·82). Addition of CT angiography spot sign (odds ratio 4·46, 95% CI 2·95–6·75; p<0·0001) to the model increased the C-index by 0·05 (95% CI 0·03–0·07).

**Interpretation:**

In this large patient-level meta-analysis, models using four or five predictors had acceptable to good discrimination. These models could inform the location and frequency of observations on patients in clinical practice, explain treatment effects in prior randomised trials, and guide the design of future trials.

**Funding:**

UK Medical Research Council and British Heart Foundation.

**Research in context**

**Evidence before this study**

We did a systematic review of studies of the risk of intracerebral haemorrhage growth, and associations with it, published in OVID MEDLINE (from Jan 1, 1970, to Dec 31, 2015) using a comprehensive search strategy, limited to humans, combining terms for intracerebral haemorrhage (“exp basal ganglia hemorrhage/”, “intracranial hemorrhages/”, “cerebral hemorrhage/”, “intracranial hemorrhage, hypertensive/”, and other text words) with text words suggesting growth (“expansion”, “growth”, or “enlargement”), with no language restrictions. When we updated the search to March 1, 2018, we identified reports of five new cohorts, representing a maximum of a 10% increase in the number of eligible patients compared with those from the 36 cohorts that provided individual patient data in this meta-analysis. We did not include these five new cohorts in our analyses. Intracerebral haemorrhage growth risk is known to be highest soon after intracerebral haemorrhage symptom onset, but its absolute risks over time and by baseline volume are unclear. Studies have identified several risk factors associated with intracerebral haemorrhage growth, but many associations are not consistent across studies, and the predictive values of these risk factors remain to be determined.

**Added value of this study**

Our systematic review led to the pooling of 5435 eligible patients from 36 cohorts, which is, to the best of our knowledge, the largest patient-level meta-analysis to explore the absolute risk and predictors of intracerebral haemorrhage growth. We found that the risks of growth over time and by baseline intracerebral haemorrhage volume were not linear. The sample size enabled us to model these associations with good precision and construct and validate multivariable models adjusted for 13 categorical or continuous covariates. Four predictors (time from symptom onset to baseline imaging, intracerebral haemorrhage volume on baseline imaging, antiplatelet use, and anticoagulant use) were independent predictors of intracerebral haemorrhage growth (C-index 0·78, 95% CI 0·75–0·82). Addition of information about the presence of spot sign on CT angiography to the model increased the C-index by just 0·05 (95% CI 0·03–0·07).

**Implications of all the available evidence**

Models using four or five predictors that are simple to collect had acceptable to good discrimination for predicting intracerebral haemorrhage growth, which was slightly improved by the addition of information on spot sign from CT angiography. These models could guide the monitoring of patients at risk of clinical deterioration as well as the interpretation and investigation of treatment effects in randomised trials.

## Introduction

Haemorrhagic stroke is responsible for around 11% of strokes in high-income countries but 22% of strokes in low-income and middle-income countries,^1^ where 75% of deaths due to haemorrhagic stroke occur.^2^ Spontaneous (non-traumatic) intracerebral haemorrhage is the most frequent type of haemorrhagic stroke and has the worst outcome: almost half of patients die within the first month and 80% of survivors are dependent on a caregiver.^3^

Intracerebral haemorrhage volume increases after vessel rupture and growth can continue after intracerebral haemorrhage is first diagnosed on brain imaging. Intracerebral haemorrhage growth is associated with poor clinical outcome.^4^ Therefore, immediately after confirmation of intracerebral haemorrhage diagnosis on brain imaging, accurate prediction of the risk of later intracerebral haemorrhage growth could help to target patients' monitoring, treatment and transfer to specialist care, and the design and interpretation of randomised trials of treatments to limit intracerebral haemorrhage growth.^5^

The timing of the first brain imaging done after intracerebral haemorrhage onset and the intracerebral haemorrhage volume found on imaging are two consistently identified risk factors for intracerebral haemorrhage growth, although the association of other potential risk factors has been inconsistent in many small observational studies. Interest has grown in whether a so-called spot sign due to contrast extravasation on additional angiography at the time of diagnostic imaging is a predictor of intracerebral haemorrhage growth.^6^ There are several multivariable prediction models for intracerebral haemorrhage growth,^7^, ^8^, ^9^, ^10^, ^11^ but the identified predictors have varied across models, and several have relied on CT angiography,^12^ which is not readily available in low-income and middle-income countries. Identifying more accurate predictors of intracerebral haemorrhage growth is recognised to be a research priority.^13^

Therefore, we aimed to identify the risk and predictors of acute intracerebral haemorrhage growth, develop and validate prediction models that could be used worldwide, and evaluate the added value of CT angiography.

## Methods

### Search strategy and selection criteria

We conducted a systematic review to identify studies of intracerebral haemorrhage growth that would share individual patient data for a patient-level meta-analysis of the absolute risks and predictors of intracerebral haemorrhage growth.^14^ A prespecified protocol (finalised on June 20, 2013, and not registered; appendix) guided our data collection and analyses.

One author (JF) identified potentially eligible cohorts by searching OVID MEDLINE from Jan 1, 1970, to Dec 31, 2015, using a comprehensive search strategy (appendix); hand-searching relevant studies' bibliographies; contacting authors of collaborating studies; and accessing patient-level data from eligible cohorts in the Virtual International Stroke Trials Archive. We included the largest single report of any observational or randomised cohort—regardless of language of publication—if it included at least ten eligible patients with acute intracerebral haemorrhage who had brain imaging (by CT with or without angiography or by MRI) to diagnose intracerebral haemorrhage and used a predefined protocol for repeat imaging (done regardless of clinical need), which would minimise the risks of selection and information biases about intracerebral haemorrhage growth.

We included patients from these cohorts if they were aged 18 years or older; had non-traumatic intracerebral haemorrhage that was probably due to cerebral small vessel disease and not secondary to an underlying structural cause identified by brain imaging; had data available from brain imaging initially done 0·5–24 h and repeated fewer than 6 days after symptom onset; had baseline intracerebral haemorrhage volume of less than 150 mL; and did not undergo acute treatment that might reduce intracerebral haemorrhage volume (ie, surgical evacuation,^15^ haemostatic therapy,^5^ or blood pressure lowering^16^). We excluded patients if the time from symptom onset to baseline imaging was not known in hours or if they had not been included in the published report of their cohort.

We emailed our protocol and an invitation to collaborate to the corresponding authors of cohorts that were eligible for inclusion, followed by one reminder. We included cohorts if corresponding authors of studies reporting them confirmed their eligibility and provided patient-level data on eligibility criteria and other variables at baseline, information on type and timing of baseline and repeat brain imaging, intracerebral haemorrhage characteristics (location, volume on baseline and repeat imaging, presence of intraventricular haemorrhage), and the presence of the spot sign on CT angiography if done (appendix).

Research ethics committees or other entities overseeing the use of patients' data had approved the collaborating cohorts. Cohorts shared only anonymised data, so neither individual consent nor specific approval for this individual patient data meta-analysis were required.

### Data analysis

We used reports of the included cohorts to categorise their method of intracerebral haemorrhage volume measurement as a cohort-level characteristic into either the manual ABC/2 method^17^ or an automated or semi-automated planimetric method.^18^ We assessed risk of bias across cohorts by identifying the studies that did not meet our eligibility criteria, did not share data, or did not provide data on a sufficient number of the variables of interest (appendix). We checked data completeness and consistency within each cohort and resolved any queries directly with the relevant collaborators. We standardised the format, coding, and units of measurement of variables to maximise the number available for analysis in all cohorts. We did not use or request aggregate data from cohorts that did not share patient-level data.

We prespecified that the primary outcome measure of intracerebral haemorrhage growth would be an increase in intracerebral haemorrhage volume between baseline and repeat imaging of more than 6 mL; we chose an absolute measure of intracerebral haemorrhage growth in volume because such measures seem to have higher positive predictive values for more severe clinical outcomes than does the combination of absolute or relative increases in intracerebral haemorrhage volume (eg, >33%).^19^

We prespecified the variables that might be predictors of intracerebral haemorrhage growth in our protocol (appendix) on the basis of their clinical relevance, likelihood of being associated with outcome, and reliability and accuracy of measurement (appendix). To these variables, we added history of liver disease and history of stroke; we also added CT angiography spot sign in view of the increasing interest in its role as a predictor since the protocol had originally been written (appendix).^6^ Of these prespecified variables, we selected potential predictors on the basis of their completeness and availability at the time of diagnosis in the available cohorts and the extent to which their selection maximised the total sample size available for multivariable analyses. Many cohorts excluded patients taking anticoagulant therapy at onset and only a few cohorts conducted CT angiography, so we took a hierarchical approach to investigating univariable and multivariable associations and predictors of intracerebral haemorrhage growth.

First, we analysed patients not taking anticoagulant therapy at intracerebral haemorrhage symptom onset because they constituted the vast majority of the included cohorts. In this dataset, we examined the associations between intracerebral haemorrhage growth and a subset of the variables, which were chosen on the basis of their completeness and availability at the time of intracerebral haemorrhage diagnosis in the participating cohorts. We visually inspected plots of cohort-specific estimates of association for each variable to exclude major heterogeneity. We then used a one-stage approach to meta-analysis to obtain unadjusted and adjusted estimates pooled across the cohorts using logistic regression models with random intercepts and random coefficients. For all continuous predictors, we used either a linear term or, where there was strong evidence (p<0·01) of non-linearity on the log-odds scale, a fractional polynomial. We described the univariable associations between intracerebral haemorrhage growth and two of the continuous variables (time to baseline imaging and intracerebral haemorrhage volume at baseline) by plotting the predicted probability of intracerebral haemorrhage growth derived from the model against the predictor. For the remaining continuous variables, we quantified the unadjusted and adjusted associations using the odds ratio for the upper quartile compared with the lower quartile based on the fitted linear or fractional polynomial terms in the logistic regression model. We had a sufficient sample size to split those patients who were not taking anticoagulant therapy by contributing cohort into two datasets: one to develop a prediction model and another to validate its performance. We did this temporal validation with patients from earlier cohorts (1994–2007) allocated to the development dataset and patients from more recent cohorts (2008–15) allocated to the validation dataset. We chose a subset of potential predictors for entry into a multivariable model on the basis of their combined availability in the development dataset and the number of patients with intracerebral haemorrhage growth (to avoid overfitting), without considering the results of the unadjusted and adjusted associations between each predictor and intracerebral haemorrhage growth. We did not examine interactions between other covariates and these associations. We derived a prediction index for intracerebral haemorrhage growth with the predictors that remained in a multivariable logistic regression model after backwards elimination. We assessed the performance of the prediction model using calibration plots of predicted versus observed probabilities, receiver operating characteristic curves, and the C-index to assess discrimination in both the development and validation datasets and in patients from cohorts that included patients taking anticoagulant therapy at intracerebral haemorrhage onset.

Second, we assessed the performance of the prediction model in patients taking anticoagulant therapy at intracerebral haemorrhage onset.

Third, we split by contributing cohort those patients from cohorts that included at least some patients taking anticoagulant therapy at intracerebral haemorrhage onset into one dataset to develop a prediction model and another to validate its performance (using temporal validation, as described above). We considered the same subset of potential predictors as for the first prediction model, with the addition of anticoagulant therapy use at intracerebral haemorrhage onset. We derived a prediction index for intracerebral haemorrhage growth and assessed its performance using the same approaches as for the first prediction model.

Fourth, in cohorts that included at least some patients with data available on the spot sign identified by CT angiography and that also included and distinguished patients taking anticoagulant therapy at onset, we assessed whether spot sign presence was independently associated with intracerebral haemorrhage growth and the predictive performance when it was added to the predictors in the second prediction model.

We did a prespecified sensitivity analysis to compare our findings using a definition of intracerebral haemorrhage growth as an absolute increase of more than 6 mL versus an absolute increase of more than 6 mL or a relative increase of more than 33% in intracerebral haemorrhage volume. We did post-hoc sensitivity analyses to compare associations between time from intracerebral haemorrhage symptom onset to baseline brain imaging and intracerebral haemorrhage volume on baseline imaging with intracerebral haemorrhage growth in cohorts using ABC/2 versus planimetric methods of measuring intracerebral haemorrhage volume and in cohorts from earlier versus later time periods.

Analyses were done using SAS software version 9.4 (SAS Institute) and Stata version 12.1 (StataCorp).

### Role of the funding source

The study sponsors had no role in study design; in the collection, analysis, and interpretation of data; in the writing of the report; and in the decision to submit the paper for publication. The data were available to all authors on request. The corresponding author had final responsibility for the decision to submit for publication.

## Results

**Figure 1:**
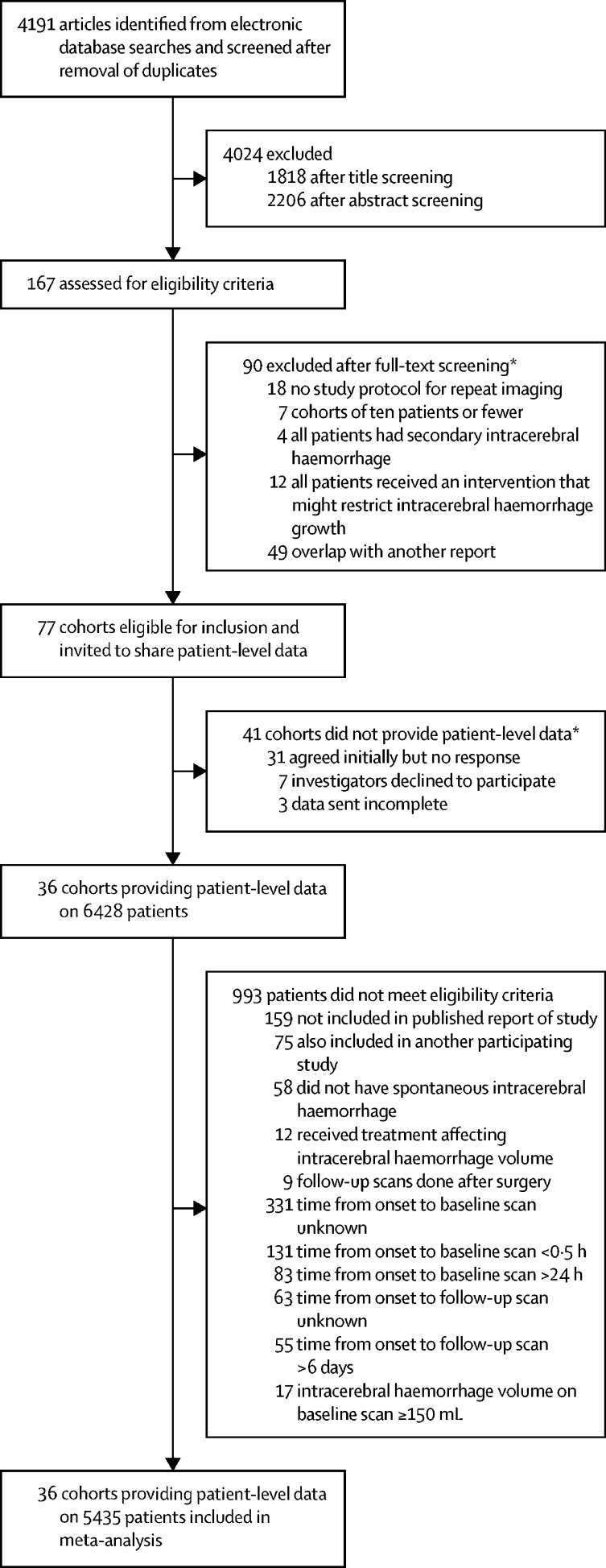
Study selection *Excluded studies and cohorts are listed in the appendix.

We screened 4191 studies identified by our searches, assessed 167 for eligibility, invited 77 eligible cohorts to share data, and obtained patient-level data from 36 (47%) cohorts^18^, ^19^, ^20^, ^21^, ^22^, ^23^, ^24^, ^25^, ^26^, ^27^, ^28^, ^29^, ^30^, ^31^, ^32^, ^33^, ^34^, ^35^, ^36^, ^37^, ^38^, ^39^, ^40^, ^41^, ^42^, ^43^, ^44^, ^45^, ^46^, ^47^, ^48^, ^49^, ^50^, ^51^ involving 6428 patients with repeat brain imaging after intracerebral haemorrhage between 1985 and 2015 (no data up to 1984 were obtained; figure 1; appendix).^20^, ^21^, ^22^, ^23^, ^24^, ^25^, ^26^, ^27^, ^28^, ^29^, ^30^, ^31^, ^32^, ^33^, ^34^, ^35^, ^36^, ^37^, ^38^, ^39^, ^40^, ^41^, ^42^, ^43^, ^44^, ^45^, ^46^, ^47^, ^48^, ^49^, ^50^, ^51^, ^52^, ^53^, ^54^, ^55^ Countries classified as high income by the World Bank contributed to 26 (72%) of 36 collaborating cohorts versus 30 (73%) of 41 eligible cohorts that did not collaborate. Planimetric methods of measuring intracerebral haemorrhage volume were used by 19 (53%) of 36 collaborating cohorts versus six (15%) of 41 eligible cohorts that did not collaborate.

After confirming the integrity of the data from eligible cohorts and excluding patients who were ineligible, we created a dataset of 5435 patients (appendix), from which we identified four groups of patients for further analysis: 5076 patients not taking anticoagulant therapy at intracerebral haemorrhage onset, 351 patients taking anticoagulant therapy at intracerebral haemorrhage onset, 3550 patients from cohorts that included at least some patients taking anticoagulant therapy at intracerebral haemorrhage onset, and 868 patients for whom both information about anticoagulant therapy at intracerebral haemorrhage onset and spot sign on acute CT angiography were known (table 1; appendix). The availability of potential predictors varied between the collaborating cohorts such that their overall completeness was 86% in the patients not taking anticoagulant therapy at intracerebral haemorrhage onset, 88% in the patients taking anticoagulant therapy at intracerebral haemorrhage onset, 89% in patients from cohorts that included at least some patients taking anticoagulant therapy at intracerebral haemorrhage onset, and 91% in the patients with information about anticoagulant therapy at intracerebral haemorrhage onset and spot sign on acute CT angiography. More than 80% of patients in all groups had repeat imaging done within 48 h of intracerebral haemorrhage onset and less than 2% of patients had repeat imaging done more than 4 days after intracerebral haemorrhage onset (appendix).

**Table 1:** Characteristics of patients included in the four datasets for meta-analysis

		**Not taking anticoagulant therapy (n=5076)**	**Taking anticoagulant therapy (n=351)**	**From cohorts with some patients taking anticoagulant therapy (n=3550)**	**CT angiography (n=868)**
Sex					
	Female	1971/4884 (40%)	135 (38%)	1449 (41%)	379 (44%)
	Male	2913/4884 (60%)	216 (62%)	2101 (59%)	489 (56%)
Age, years		67 (56–76)	76 (69–82)	69 (58–78)	70 (57–79)
Previous stroke		607/4560 (13%)	77/317 (24%)	481/3308 (15%)	97/829 (12%)
	Previous intracerebral haemorrhage*	179/2753 (7%)	9/246 (4%)	113/2051 (6%)	29/805 (4%)
	Previous ischaemic stroke*	246/2755 (9%)	53/246 (22%)	213/2051 (10%)	70/805 (9%)
History of hypertension		3787/5050 (75%)	291 (83%)	2739/3547 (77%)	616/866 (71%)
History of diabetes mellitus		727/4197 (17%)	82/343 (24%)	626/3475 (18%)	137/807 (17%)
History of liver disease		256/3360 (8%)	14/220 (6%)	96/1946 (5%)	15/360 (4%)
History of excessive alcohol consumption†		568/3091 (18%)	20/177 (11%)	221/1455 (15%)	73/554 (13%)
Antiplatelet therapy at symptom onset		913/5030 (18%)	102 (29%)	855/3543 (24%)	225/837 (27%)
Anticoagulant therapy at symptom onset		0	351 (100%)	349/3547 (10%)	87/841 (10%)
Systolic blood pressure at presentation, mm Hg		177 (158–198); n=4882	170 (147–190); n=320	177 (157–197); n=3333	175 (150–200); n=860
Blood glucose at presentation, mmol/L		7·0 (5·9–8·7); n=4265	7·4 (6·0–9·3); n=340	7·0 (5·9–8·7); n=3417	7·3 (6·1–8·9); n=864
Platelet count (×10^9^/L) at presentation		221 (181–266); n=3857	209 (174–260); n=289	222 (185–267); n=2284	227 (181–273); n=862
Glasgow Coma Scale score at presentation					
	3–6	285/4564 (6%)	42/342 (12%)	248/3502 (7%)	73/831 (9%)
	7–12	1033/4564 (23%)	81/342 (24%)	824/3502 (24%)	193/831 (23%)
	13–14	1157/4564 (25%)	70/342 (20%)	830/3502 (24%)	151/831 (18%)
	15	2089/4564 (46%)	149/342 (44%)	1600/3502 (46%)	414/831 (50%)
NIHSS score at presentation		12 (7–18); n=2661	13 (7–17); n=126	12 (7–17); n=2014	14 (6–18); n=325
Time from symptom onset to baseline imaging, h		2·4 (1·3–4·7)	3·3 (1·7–6·4)	2·2 (1·3–4·2)	2·9 (1·5–5·1)
Intracerebral haemorrhage volume on baseline imaging, mL		13·2 (6·3–30·0)	16·0 (6·4–39·0)	13·4 (6·6–30·3)	15·0 (6·6–34·1)
Lobar location of intracerebral haemorrhage on baseline imaging		1080/4920 (22%)	129/344 (38%)	907/3439 (26%)	267/866 (31%)
Intraventricular haemorrhage present on baseline imaging		1834/4980 (37%)	157/348 (45%)	1265/3452 (37%)	344 (40%)
CT angiogram spot sign present		..	..	..	204 (24%)
>6 mL intracerebral haemorrhage growth		1009 (20%)	110 (31%)	771 (22%)	177 (20%)
>6 mL or >33% intracerebral haemorrhage growth		1301 (26%)	139 (40%)	986 (28%)	219 (25%)

Data are n (%), n/N (%), or median (IQR). NIHSS=National Institutes of Health Stroke Scale.

* Available in a subgroup of cohorts that quantified the subtype of previous stroke. Not all cohorts that quantified the subtype included both intracerebral haemorrhage and ischaemic stroke.

† Definition of excessive consumption varied across cohorts.

**Figure 2:**
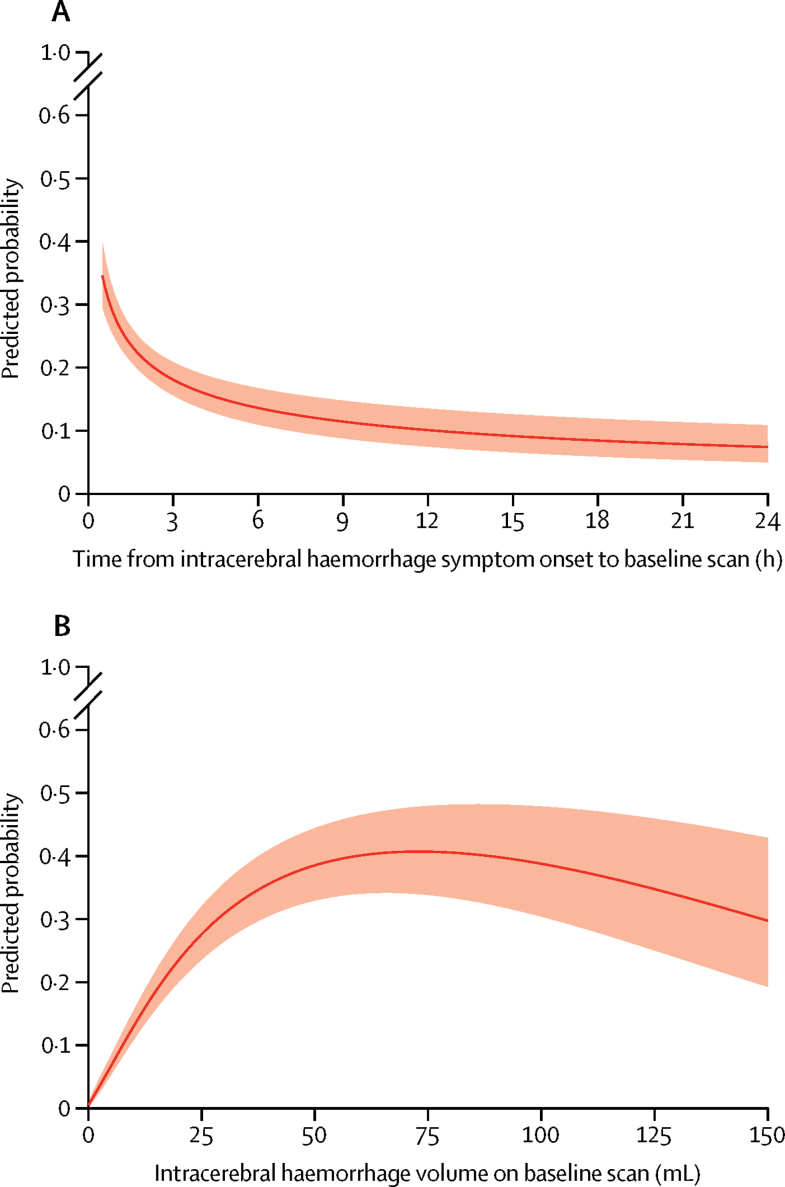
Predicted probability of intracerebral haemorrhage growth >6 mL Data calculated on 5076 patients who were not taking anticoagulant therapy at symptom onset. (A) Predicted probability by time from intracerebral haemorrhage symptom onset to baseline imaging, and (B) according to intracerebral haemorrhage volume on baseline imaging. The solid line indicates predicted probability and the shaded region indicates the 95% CIs.

When assessing the two variables with non-linear associations, we found that in patients not taking anticoagulant therapy at intracerebral haemorrhage onset, the predicted probability of intracerebral haemorrhage growth declined with increasing time from intracerebral haemorrhage symptom onset to baseline imaging: the rate of decline was steepest 0·5–3 h after intracerebral haemorrhage symptom onset (figure 2A). The predicted probability of intracerebral haemorrhage growth increased with increasing intracerebral haemorrhage volume on baseline brain imaging and peaked at about 75 mL, above which it declined (figure 2B). We aimed to quantify the associations between 17 additional variables and the occurrence of intracerebral haemorrhage growth (appendix). There were too few patients with data for six variables (previous intracerebral haemorrhage, previous ischaemic stroke, history of liver disease, history of excessive alcohol consumption, platelet count at presentation, and National Institutes of Health Stroke Scale [NIHSS] score at presentation). Therefore, we selected 13 of the 19 variables as potential predictors for a multivariable model in patients not taking anticoagulant therapy, on the basis of maximising the number of predictors being considered while also maximising the number of patients with complete data for all the predictors chosen for the subset: time from symptom onset to baseline imaging, intracerebral haemorrhage volume on baseline imaging, sex, age, previous stroke, history of hypertension, history of diabetes, antiplatelet therapy at symptom onset, systolic blood pressure at presentation, blood glucose at presentation, Glasgow Coma Scale score at presentation, intracerebral haemorrhage location on baseline scan, and intraventricular haemorrhage on baseline scan. We restricted all further analyses to datasets of patients with complete data on these 13 potential predictors.

3479 patients who were not taking anticoagulant therapy at intracerebral haemorrhage onset had data available for the 13 predictors. We developed a prediction model for intracerebral haemorrhage growth using a dataset of 2534 (73%) of these patients from 18 earlier cohorts (ie, 1994–2007; appendix). From the 13 potential predictors considered, three significant predictors constituted the final model (table 2):

$$\text{Predicted}\;\text{probability}\;\text{of}\;\text{intracerebral}\;\text{haemorrhage}\;\text{growth}=\frac{1}{\left(1+e^{-\text{PI}}\right)}$$

where the predictive index (PI) is given by

**Table 2:** Multivariable models of predictors of intracerebral haemorrhage growth >6 mL

	**Comparison**	**Odds ratio (95% CI)**	**p value**
Patients not taking anticoagulant therapy at symptom onset*			
Time from symptom onset to baseline imaging, h†	3·4 *vs* 1·2	0·65 (0·51–0·82)	0·0003
Intracerebral haemorrhage volume on baseline imaging, mL†	28 *vs* 7	4·73 (3·81–5·87)	<0·0001
Antiplatelet therapy at symptom onset	Yes *vs* no	1·38 (1·06–1·79)	0·016
**Patients from cohorts including at least some patients taking anticoagulant therapy at symptom onset**‡			
Time from symptom onset to baseline imaging, h†	3·5 *vs* 1·2	0·59 (0·42–0·82)	0·0021
Intracerebral haemorrhage volume on baseline imaging, mL†	29 *vs* 7	4·81 (3·82–6·05)	<0·0001
Antiplatelet therapy at symptom onset	Yes *vs* no	1·36 (1·04–1·78)	0·023
Anticoagulant therapy at symptom onset	Yes *vs* no	2·91 (1·97–4·26)	<0·0001

* Data were calculated on 2534 patients from 18 cohorts (appendix).

† The odds ratios for time from symptom onset to baseline imaging and intracerebral haemorrhage volume on baseline imaging are for upper quartile compared with lower quartile.

‡ Data were calculated on 2381 patients from ten cohorts (appendix).

−4·254−0·196time−0·0754volume+1·186√volume+0·320antiplatelet

with time measured in hours, volume measured in mL, and antiplatelet an indicator variable for antiplatelet therapy at intracerebral haemorrhage onset taking values 1 for yes and 0 for no.

This first prediction model had good calibration (appendix) and its discrimination was good in both the development dataset (C-index 0·75, 95% CI 0·72–0·77) and the temporal validation dataset of 945 (27%) patients from six later cohorts (0·76, 0·73–0·79). This prediction model, derived in patients who were not taking anticoagulant therapy at symptom onset, underestimated the probability of intracerebral haemorrhage growth in the 351 patients in 21 cohorts who were taking anticoagulant therapy at symptom onset (appendix), but its discrimination remained good (0·73, 0·68–0·79).

We also developed a prediction model for intracerebral haemorrhage growth using a dataset of 2381 patients from ten cohorts that included at least some patients taking anticoagulant therapy at intracerebral haemorrhage onset (appendix). From the 13 potential predictors plus anticoagulant therapy at intracerebral haemorrhage symptom onset, four predictors constituted the final model (table 2), where PI is given by

−4·426−0·230time   −0·0776volume+1·196√volume   +0·310antiplatelet+1·065anticoagulant

where anticoagulant is an indicator variable for anticoagulant therapy at intracerebral haemorrhage onset taking values 1 for yes and 0 for no.

This second prediction model was well calibrated (appendix) and its discrimination was good in both the development dataset (C-index 0·75, 95% CI 0·73–0·78) and the validation dataset of 895 patients from five cohorts (0·74, 0·71–0·78).

Finally, to assess the additional predictive value of spot sign on CT angiography, we assessed the performance of a third prediction model in the 837 patients from six cohorts with available data on all covariates (appendix), where PI is given by

−4·954−0·138time−0·0769volume   +1·139√volume+0·370antiplatelet   +1·028anticoagulant+1·496spot

where spot is an indicator variable for presence of CT angiography spot sign taking values 1 for present and 0 for absent.

**Figure 3:**
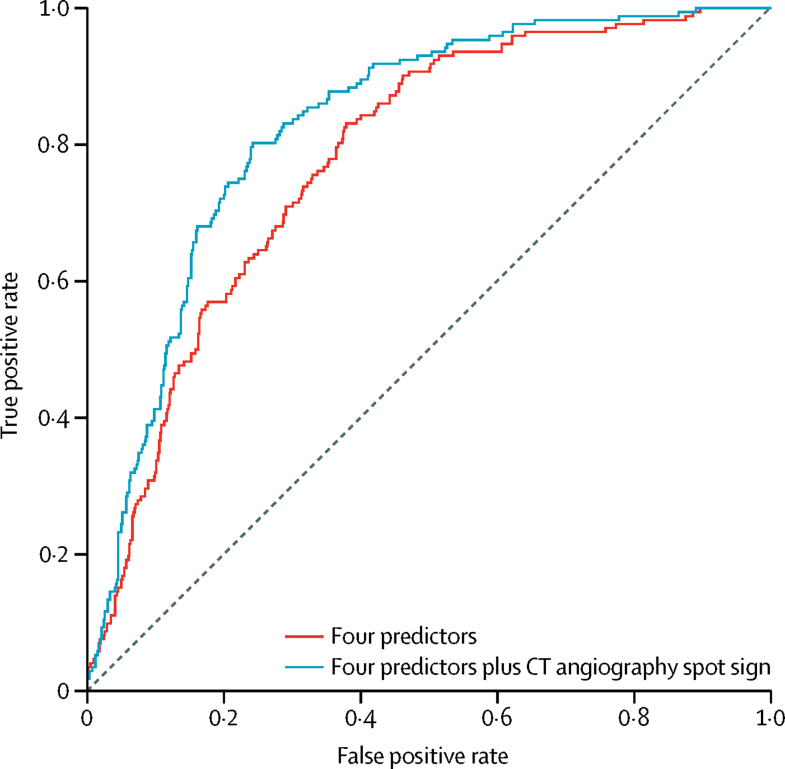
Receiver operating characteristic curves for the predicted probability of intracerebral haemorrhage growth >6 mL Data calculated on 837 patients with assessment of CT angiography spot sign, data on antiplatelet therapy, and data on anticoagulant therapy use at symptom onset. Receiver operating characteristic curves used four predictors (time from symptom onset to baseline imaging [h], intracerebral haemorrhage volume on baseline imaging [mL], antiplatelet therapy at symptom onset, and anticoagulant therapy at symptom onset) and four predictors plus CT angiography spot sign.

The presence of the spot sign was strongly and independently associated with the occurrence of intracerebral haemorrhage growth (table 3) and improved the C-index of the prediction model by 0·05 (95% CI 0·03–0·07) from 0·78 (0·75–0·82) to 0·83 (0·80–0·86; figure 3).

**Table 3:** Multivariable models of predictors of intracerebral haemorrhage growth >6 mL in patients with assessment of CT angiography spot sign, data on antiplatelet therapy, and data on anticoagulant therapy use at symptom onset

	**Comparison**	**Four predictors**		**Four predictors with the addition of CT angiography spot sign**	
		Odds ratio (95% CI)	p value	Odds ratio (95% CI)	p value
Time from symptom onset to baseline imaging, h*	5·1 *vs* 1·5	0·50 (0·36–0·70)	<0·0001	0·61 (0·44–0·84)	0·0030
Intracranial haemorrhage volume on baseline imaging, mL*	33 *vs* 6	7·18 (4·46–11·56)	<0·0001	5·35 (3·25–8·81)	<0·0001
Antiplatelet therapy at symptom onset	Yes *vs* no	1·68 (1·06–2·66)	0·026	1·45 (0·89–2·35)	0·13
Anticoagulant therapy at symptom onset	Yes *vs* no	3·48 (1·96–6·16)	<0·0001	2·80 (1·53–5·10)	0·0008
CT angiography spot sign	Present *vs* absent	..	..	4·46 (2·95–6·75)	<0·0001

Data were calculated on 837 patients from six cohorts (appendix).

* Odds ratios for time from symptom onset to baseline imaging and intracranial haemorrhage volume on baseline imaging are for upper quartile *vs* lower quartile.

We assessed the performance of the second and third prediction models at different thresholds of predicted probability of intracerebral haemorrhage growth and found very few significant differences in sensitivity, specificity, positive predictive value, and negative predictive value (appendix).

In a prespecified sensitivity analysis, when we defined intracerebral haemorrhage growth as an absolute increase of more than 6 mL or a relative increase of more than 33% in intracerebral haemorrhage volume between baseline and follow-up imaging, the direction, strength, and significance of the adjusted associations between almost all predictors and intracerebral haemorrhage growth remained the same (appendix), and the C-index of our second prediction model improved from 0·71 (95% CI 0·67–0·75) to 0·76 (0·72–0·80) with the addition of information from CT angiography (appendix). In a post-hoc sensitivity analysis, we found no evidence that the risk of intracerebral haemorrhage growth according to time from symptom onset to baseline imaging or according to intracerebral haemorrhage volume on baseline imaging differed by cohort epoch or volumetric method used (appendix).

## Discussion

This collaborative meta-analysis evaluated 19 covariates in one or more analyses of predictors of intracerebral haemorrhage growth from 5435 eligible patients in 36 cohorts. We identified novel non-linear associations between the probability of intracerebral haemorrhage growth and both the time from symptom onset to baseline imaging and baseline intracerebral haemorrhage volume. We showed that only four predictors that are simple to collect (time from symptom onset to baseline imaging, intracerebral haemorrhage volume on baseline imaging, antiplatelet use, and anticoagulant use) were independently associated with intracerebral haemorrhage growth in multivariable models, and a prediction model that we developed using these predictors not only had good calibration and discrimination but also done well in an external validation dataset. The addition of information about the presence of spot sign on CT angiography to this prediction model gave a small increase in discrimination.

Although many studies have investigated unadjusted and adjusted associations between a wide variety of clinical, blood, genetic, imaging, and pharmacological factors and the occurrence of intracerebral haemorrhage growth, only a few prediction models have been developed and the predictors used have varied considerably.^7^, ^8^, ^9^, ^10^, ^11^, ^51^ Since 2011, there has been growing interest in use of the spot sign on CT angiography for predicting intracerebral haemorrhage growth,^10^, ^30^ but the clinical utility of the small increase in discrimination that resource-intensive advanced vascular imaging adds to simple clinical and imaging predictors that are available worldwide is unclear.

The strengths of this study include its large sample size and availability of many predictors from geographically diverse cohorts to develop and externally validate prediction models involving simple predictors that could be used in any health-care setting, as well as the added value of CT angiography in high-income countries. We minimised the risk of selection and information biases by restricting eligibility to cohorts that had defined when they would repeat brain imaging soon after intracerebral haemorrhage onset in all survivors and not according to clinical need alone.

Although our study was large, only half of the investigators of the available cohorts shared patient-level data. Most cohorts were assembled in high-income countries. A shortage of data on the following variables precluded their inclusion in our prediction models: previous intracerebral haemorrhage, previous ischaemic stroke, history of liver disease, history of excessive alcohol consumption, platelet count at presentation, and NIHSS score at presentation. Since the end of the literature search that defined inclusion in our analyses, our update of the search to March 1, 2018, identified reports of five new cohorts involving 669 patients, representing a maximum of a 10% increase over the 6428 patients from 36 cohorts that provided individual patient data. Nonetheless, the sample size we achieved allowed us to develop and validate prediction models using a large number of widely available predictors, without omitting any predictors that had been identified by previous prediction models. Included cohorts with data collected in the 1990s might not have used multiple-row detector array technology and digitisation, which might have affected their accuracy of intracerebral haemorrhage volume measurement, although there was no evidence that our findings differed by cohort epoch in sensitivity analyses. 19 (53%) of 36 cohorts used planimetric methods to estimate intracerebral haemorrhage volume but 17 (47%) of 36 cohorts used the ABC/2 method (which can marginally overestimate intracerebral haemorrhage volume^18^), although we found no evidence that our findings differed by volumetric method in sensitivity analyses. Since these cohorts were studied, a variety of new imaging signs (eg, density, irregularity, fluid level, hypodensity, island, satellite, swirl,^56^ blend,^37^ and black hole^57^) have been described, but we were unable to evaluate them because they were not collected by the collaborating cohorts and we could not re-evaluate patients' imaging. However, our simple prediction models provide the basis upon which the added value of these new signs can be assessed, as we have done for the CT angiography spot sign.

We found that the rate of decline in the probability of intracerebral haemorrhage growth was steepest during the 0·5–3 h after intracerebral haemorrhage symptom onset and that the predicted probability of intracerebral haemorrhage growth peaked at an intracerebral haemorrhage volume of about 75 mL. These findings could in part explain the neutral results of recent randomised trials of acute interventions designed to limit intracerebral haemorrhage growth, which enrolled many patients towards or beyond the time of greatest risk of intracerebral haemorrhage growth and most patients had small intracerebral haemorrhages at low probability of growth. For example, the average time to randomisation after intracerebral haemorrhage symptom onset and average intracerebral haemorrhage volume were 3·7 h and 13 mL in TICH2,^58^ 3·7 h and 11 mL in INTERACT2,^22^ 3·1 h and 10 mL in ATACH2,^59^ and 2·7 h and 22–24 mL in FAST.^40^ In particular, our findings about the association between time after intracerebral haemorrhage symptom onset and the probability of intracerebral haemorrhage growth emphasise the importance of extremely rapid assessment, investigation, and randomisation in future trials of therapies to improve outcome by limiting intracerebral haemorrhage growth.

The prediction models that we have developed could be useful in clinical practice for predicting the risk of intracerebral haemorrhage growth, which is recommended in the emergency assessment of acute intracerebral haemorrhage. The clinically useful threshold for the predicted probability of intracerebral haemorrhage growth will vary according to its desired accuracy (appendix), the clinical setting, and future therapeutic advances, such that our models might help in determining patients' place of care and frequency of observation.^60^
