# Corrections

**DOI:** 10.1016/S1474-4422(18)30354-5

**Published:** 2018-11

*Al-Shahi Salman R, Frantzias J, Lee RJ, et al. Absolute risk and predictors of the growth of acute spontaneous intracerebral haemorrhage: a systematic review and meta-analysis of individual patient data.* Lancet Neurol *2018; **17**: 885–94*—The affiliation of Department of Neurology, University of Erlangen-Nuremberg, Erlangen, Germany has been added for authors Dimitre Staykov and Bastian Volbers. This correction has been made to the online version as of Sept 19, 2018.

