# Supplementary material for: Absolute risk and predictors of the growth of acute spontaneous intracerebral haemorrhage: a systematic review and meta-analysis of individual patient data
Source: Lancet Neurol. 2018 Oct;17(10):885–94. doi: 10.1016/S1474-4422(18)30253-9 (PMC6143589; doi:10.1016/S1474-4422(18)30253-9)
Supplement: Supplementary appendix [file mmc1.pdf]

THE LANCET
Neurology

Supplementary appendix

This appendix formed part of the original submission and has been peer reviewed. We post it as supplied by the authors.

Supplement to: Al-Shahi Salman R, Frantzias J, Lee RJ, et al, for the VISTA-ICH Collaboration and the ICH Growth Individual Patient Data Meta-analysis Collaborators. Absolute risk and predictors of the growth of acute spontaneous intracerebral haemorrhage: a systematic review and meta-analysis of individual patient data. *Lancet Neurol* 2018; published online August 14. http://dx.doi.org/10.1016/S1474-4422(18)30253-9

Protocol (20 June 2013)

The absolute risk of acute spontaneous intracerebral haemorrhage growth over time and its determinants: individual patient data meta-analysis

Investigators

Dr Joseph Frantzias, Academic foundation doctor, NHS Lothian.
Mr Robert Lee, Research fellow, Centre for Population Health Sciences, University of Edinburgh.
Prof Rustam Al-Shahi Salman, MRC senior clinical fellow, University of Edinburgh; Honorary consultant neurologist, NHS Lothian.

Background

Spontaneous (non-traumatic) intracerebral haemorrhage (ICH) is responsible for 10-15% of strokes in developed countries, almost half of the patients die within the first month, and 80% of the survivors are dependent [1]. Organised inpatient care on stroke units improves outcome after ICH [2]. Outcome may also be improved by acute blood pressure lowering [3] and neurosurgical evacuation of ICH [4, 5], potentially by limiting ICH growth.

Initial ICH volume and the occurrence of haematoma growth have both been shown to be powerful determinants of outcome after ICH [6,7]. A number of studies have shown that any ICH growth (defined as any increase in parenchymal volume or intraventricular extension) occurs in >70% of patients scanned within three hours of ICH onset, and up to 26% of patients have significant (defined as >33%) increase in haematoma volume within 24 hours of ICH onset [8-12], which is associated with early neurological deterioration. As a result, the identification of techniques to predict ICH growth was set as one of the research priorities by a NINDS Workshop and others [13,14]: this could be most easily achieved by developing statistical prediction models of the risk of ICH growth over the entire time course of haematoma expansion using simple clinical variables.

Although the risk of ICH growth appears to be highest in patients who are first scanned within three hours of ICH onset, the risk of ICH growth is not so well defined in patients who are first scanned later, despite a number of studies suggesting that bleeding can occur for several hours after onset [15,16]. Knowing the risk of ICH growth in patients who are first scanned later than three hours after ICH onset would also help better define the time window for randomized-controlled trials aiming to improve ICH outcome by limiting haematoma growth.

Thus far, the most reliable predictors of ICH growth appear to be time from ICH symptom onset to baseline CT [7,8,11], and large baseline ICH volume [12]. Many other predictors of haematoma growth have been sought in other observational studies and trials [17-20], but individual predictors have seldom been examined in more than one study, studies have not been consistent in their findings as to which of these predictors are significant [20], studies did not have sufficient power to study several predictors in their sample [21], and these studies have differed in their definitions of haematoma growth, timings of baseline and repeat scans and baseline patient characteristics.

Because these differences between studies preclude meta-analysis, we plan to use the technique of individual patient data meta-analysis to generate sufficient power to further explore the absolute risk of ICH growth over time after ICH onset, to explore potential predictors of ICH growth, and to develop a simple prediction algorithm for ICH growth [21].

Hypothesis

Our hypothesis is that haematoma growth continues to occur later than three hours after symptom onset, and that - in addition to time from onset - demographics, comorbidities, and drugs also influence its occurrence.

Methods

Research design
An individual patient meta-analysis will allow full exploration of the magnitude, frequency, and predictors of ICH growth (using a consistent definition applied to individual patients' absolute ICH volumes) over time following ICH symptom onset [21].

Identification of studies
We have used a variety of electronic databases, and hand searching pertinent studies' bibliographies, to identify studies potentially eligible for our meta-analysis. Their eligibility based on the inclusion and exclusion criteria below will be decided by the three main investigators (RS, JF, RL) on the basis of studies' publications, with further information requested from studies' authors if required. Some of these studies are randomised controlled trials whose individual

patient data are curated within VISTA-ICH; other trials and observational studies are not within VISTA-ICH (and some of these studies' authors have already contributed their datasets to this collaborative project).

Inclusion and exclusion criteria

Inclusion criteria
- Acute spontaneous (non-traumatic) intracerebral haemorrhage confirmed by brain imaging (CT or MRI).
- Repeat brain imaging (CT or MRI) performed after the first brain imaging study.

Exclusion criteria
- Age less than 18 years.
- Secondary causes of ICH (including trauma, tumour, intracranial aneurysm, arteriovenous malformation, arteriovenous fistula, cavernous malformation)
- Haemorrhagic transformation of ischaemic stoke.
- Exclusively intraventricular/subarachnoid/subdural/extradural haemorrhage.
- Studies reporting patients who are included in other publications that reported a larger cohort.
- Patients in the treatment arm of studies where participants were treated with interventions that have been shown to limit ICH growth (i.e. recombinant activated factor VII, acute blood pressure lowering, and surgery including stereotactic aspiration). Please note that we are only interested in the placebo arm of these studies.
- Studies where the decision for repeat imaging was based on clinical parameters only.
- Patients who could potentially be eligible for inclusion in our meta-analysis, but whose data have not been published in the specific study.

Data collected
The following data will be collected for each patient: sex, age at presentation, whether on anticoagulant/antiplatelet therapy at symptom onset, blood pressure and mean arterial pressure, GCS and/or NIHSS at presentation, any history of ischaemic stroke or ICH, history of hypertension/diabetes mellitus/excessive alcohol consumption, blood glucose, fibrinogen, platelet count and serum cholesterol at presentation, nature of symptom onset (awoke from sleep, last seen well, or awake at onset); time from symptom onset to first scan, type of first scan, location of ICH, whether multiple bleeds are present, morphology of ICH, ICH volume on first scan, intraventricular extension on first scan, time from symptom onset to second scan, ICH volume on second scan, type of second scan, occurrence and time of death.

Statistical analysis
1. For each dataset, we will check the completeness, ranges, and values of the data provided. We will require the same dataset that has already been published.
2. We will standardize the format and coding of the variables across the collaborating studies.
3. Using the individual patient data from all collaborating studies, we will quantify the absolute risk of ICH growth in several time windows corresponding to when the first scan was performed after ICH onset stratified by the timing of the patients' second scans), and explore potential predictors of ICH growth in univariate analyses, moving on to multivariable analyses if appropriate and there is sufficient power in the dataset.
4. Using the individual patient data from all collaborating studies, we aim to develop a prediction model for ICH growth.

Transfer of data and confidentiality
The preferred formats for data transfer are the following: Excel spreadsheet, Access database, SAS transport file, SPSS portable file, delimited text file, fixed-format text file. Authors are asked to ensure anonymisation of the data before transfer by removing all patient-identifiable data, and replacing them with a unique study ID number for each patient. All data sent are held securely in the strictest confidence.

Publication policy

We intend to include and order the authors according to (a) their contribution to the design and execution of this study and (b) the number of eligible patients they contribute with a complete dataset. The VISTA-ICH collaboration will be listed as a group author name. We will share the results of our analyses with participating groups. All authors will have the opportunity to review the manuscript and approve the final version for submission to a journal.

References

1. van Asch CJ, et al. Incidence, case fatality, and functional outcome of intracerebral haemorrhage over time, according to age, sex, and ethnic origin: a systematic review and meta-analysis. Lancet Neurology 2010; 9(2):167-176.

2. Stroke Unit Trialists' Collaboration. Organised inpatient (stroke unit) care for stroke. Cochrane Database Syst Rev 2007;(4):CD000197.
3. Anderson CS, Heeley E, Huang Y, Wang J, Stapf C, Delcourt C, Lindley R, Robinson T, Lavados P, Neal B, Hata J, Arima H, Parsons M, Li Y, Wang J, Heritier S, Li Q, Woodward M, Simes RJ, Davis SM, Chalmers J; the INTERACT2 Investigators. Rapid Blood-Pressure Lowering in Patients with Acute Intracerebral Hemorrhage. N Engl J Med 2013 [Epub ahead of print]
4. Prasad K, Mendelow AD, Gregson B. Surgery for primary supratentorial intracerebral haemorrhage. Cochrane Database Syst Rev 2008;(4):CD000200.
5. Gregson BA, Broderick JP, Auer LM, Batjer H, Chen XC, Juvela S, Morgenstern LB, Pantazis GC, Teernstra OP, Wang WZ, Zuccarello M, Mendelow AD. Individual patient data subgroup meta-analysis of surgery for spontaneous supratentorial intracerebral hemorrhage. Stroke 2012;43(6):1496-504
6. Davis SM, Broderick J, Hennerici M, Brun NC, Diringer MN, Mayer SA, Begtrup K, Steiner T, for the Recombinant Activated Factor VII Intracerebral Hemorrhage Trial Investigators. Hematoma growth is a determinant of mortality and poor outcome after intracerebral hemorrhage. Neurology, 2006;66:1175.
7. Brott, T., J. Broderick, et al. Early hemorrhage growth in patients with intracerebral hemorrhage. Stroke 1997; 28(1): 1-5.
8. Fujii Y., et al. Multivariate analysis of predictors of hematoma enlargement in spontaneous intracerebral hemorrhage. Stroke 1998;29:1160.
9. Fujitsu K., et al. Indications for surgical treatment of putaminal hemorrhage. Comparative study based on serial CT and time-course analysis. J Neurosurg 1990;73:518.
10. Kazui S., et al. Enlargement of spontaneous intracerebral hemorrhage. Incidence and time course. Stroke 1996;27:1783.
11. Kazui S., et al. Predisposing factors to enlargement of spontaneous intracerebral hematoma. Stroke 1997;28:2370.
12. Broderick JP., et al. Determinants of intracerebral hemorrhage growth: an exploratory analysis. Stroke 2007; 38:1072.
13. NINDS ICH Workshop Participants. Priorities for clinical research in intracerebral hemorrhage: report from a National Institute of Neurological Disorders and Stroke Workshop. Stroke 2005; 36(3):e23-41.
14. Steiner T., Petersson J., et al. European research priorities for intracerebral haemorrhage. Cerebrovasc Dis 2011;32(5):409-19.
15. Morgenstern LB., et al. Rebleeding leads to poor outcome in ultra-early craniotomy for intracerebral hemorrhage. Neurology 2001; 56:1294.
16. Kaneko M., et al. Long-term evaluation of ultra-early operation for hypertensive intracerebral hemorrhage in 100 cases. J Neurosurg 1983; 58:838.
17. Silva., et al. Molecular signatures of vascular injury are associated with early growth of intracerebral hemorrhage. Stroke 2005; 36:86-91.
18. Leira R., et al. Early neurologic deterioration in intracerebral hemorrhage: predictors and associated factors. Neurology 2004;63:461-467.
19. Delgado P., et al. Plasma d-dimer predicts poor outcome after acute intracerebral hemorrhage. Neurology 2006; 67:94-98.
20. Steiner T., Bosel J. Options to restrict hematoma expansion after spontaneous intracerebral hemorrhage. Stroke 2010: 41:402-9.
21. Riley RD., et al. Meta-analysis of individual participant data: rationale, conduct, and reporting. BMJ 2010; 340:521.

OVID Medline search strategy

1. exp basal ganglia hemorrhage/
2. intracranial hemorrhages/
3. cerebral hemorrhage/
4. intracranial hemorrhage, hypertensive/
5. ((brain$ or cerebr$ or intracerebral or intracran$ or parenchymal or intraventricular or infratentorial or supratentorial or basal gangli$ or putaminal or putamen or posterior fossa or neuron$ or nervous) adj10 (haemorrhage$ or hemorrhage$ or haematoma$ or hematoma$ or bleed)).tw.
6. Or/1-5
7. Humans/
8. Expansion.tw
9. Growth.tw
10. Enlargement.tw
11. Or/8-10
12. 6 AND 7 AND 11

Variables requested on each patient from studies approached for collaboration

Initial request

1. Time from symptom onset to first CT scan (specify units/categories, and how wake-up stroke was handled)
2. ICH volume on first CT scan (specify units/categories)
3. Sex of patient (specify codes used for female and male)
4. Age of patient at presentation (years)
5. Anticoagulant therapy at symptom onset (specify categories and codes, or whether an exclusion criterion)
6. Antiplatelet therapy at symptom onset (specify categories and codes, or whether an exclusion criterion)
7. Systolic blood pressure at presentation (mmHg)
8. Diastolic blood pressure at presentation (mmHg)
9. Mean arterial pressure at presentation (mmHg)
10. Glasgow Coma Scale score at presentation (details, and provide eye/verbal/motor breakdown if possible)
11. NIHSS score at presentation
12. Past history of ischaemic stroke (specify categories and codes)*
13. Past history of intracerebral haemorrhage (specify categories and codes)*
14. Past history of hypertension (specify how defined, categories and codes)
15. Past history of diabetes mellitus (specify categories and codes)
16. Past history of excessive alcohol consumption (specify how defined, categories and codes)
17. Blood glucose at presentation (specify units/categories)
18. Fibrinogen at presentation (specify units/categories)
19. Platelet count at presentation (specify units/categories)
20. Serum cholesterol at presentation (specify units/categories)
21. Location of ICH (specify, ideally: lobar, deep, brainstem, cerebellum)
22. Single or multiple ICH (specify categories and codes)
23. Morphology of ICH (specify categories and codes)
24. Intraventricular haemorrhage on first CT scan (specify units/categories)
25. Time from symptom onset to latest CT scan before any surgery (specify units/categories)
26. ICH volume on latest CT scan before any surgery (specify units/categories)

** some studies could only provide 'history of stroke' (without specifying sub-type), so in studies where stroke sub-typing was available these two variables were combined into a 'history of stroke' variable to maximise the number of studies contributing to the analysis*

Follow-up request

27. History of liver disease
28. Conduct of CT angiography
29. Presence/absence of 'spot sign' on CT angiography

References to excluded studies

A. Studies excluded

i. No study protocol for repeat imaging

1. Bae HG, Lee KS, Yun IG, et al. Rapid expansion of hypertensive intracerebral hemorrhage. Neurosurgery 1992; 31: 35-41.
2. Chan S, Conell C, Veerina KT, Rao VA, Flint AC. Prediction of intracerebral haemorrhage expansion with clinical, laboratory, pharmacologic, and noncontrast radiographic variables. *Int J Stroke* 2015; 10: 1057-1061.
3. Fehr MA, Anderson DC. Incidence of progression or rebleeding in hypertensive intracerebral hemorrhage. J Stroke Cerebrovasc Dis 1991; 1: 111-116.
4. Herold S, von Kummer R, Jaeger C. Follow-up of spontaneous intracerebral haemorrhage by computed tomography. *J Neurol* 1982; 228: 267-276.
5. Kim J, Smith A, Hemphill JC 3rd, et al. Contrast extravasation on CT predicts mortality in primary intracerebral hemorrhage. AJNR Am J Neuroradiol 2008; 29: 520-525.
6. Lou M, Al-Hazzani A, Goddeau RP Jr, Novak V, Selim M. Relationship between white-matter hyperintensities and hematoma volume and growth in patients with intracerebral hemorrhage. Stroke 2010; 41: 34-40.
7. Mayer SA, Sacco RL, Shi T, Mohr JP. Neurologic deterioration in noncomatose patients with supratentorial intracerebral hemorrhage. Neurology 1994; 44: 1379-1384.
8. Naidech AM, Maas MB, Levasseur-Franklin KE, Liotta EM, Guth JC, Berman M, Rosenow JM, Lindholm PF, Bendok BR, Prabhakaran S, Bernstein RA, Kwaan HC. Desmopressin improves platelet activity in acute intracerebral hemorrhage. *Stroke* 2014; 45: 2451-2453.
9. Naidech AM, Maas MB, Levasseur-Franklin KE, Liotta EM, Guth JC, Berman M, Rosenow JM, Lindholm PF, Bendok BR, Prabhakaran S, Bernstein RA, Kwaan HC. Desmopressin improves platelet activity in acute intracerebral hemorrhage. *Stroke* 2014; 45: 2451-2453.
10. Qureshi AI, Mohammad YM, Yahia AM, et al. A prospective multicenter study to evaluate the feasibility and safety of aggressive antihypertensive treatment in patients with acute intracerebral hemorrhage. J Intensive Care Med 2005; 20: 34-42.
11. Saloheimo P, Ahonen M, Juvela S, Pyhtinen J, Savolainen ER, Hillbom M. Regular aspirin-use preceding the onset of primary intracerebral hemorrhage is an independent predictor for death. Stroke 2006; 37: 129-133.
12. Sansing LH, Kaznatcheeva EA, Perkins CJ, Komaroff E, Gutman FB, Newman GC. Edema after intracerebral hemorrhage: correlations with coagulation parameters and treatment. J Neurosurg 2003; 98, 985-992.
13. Schindlbeck KA, Santaella A, Galinovic I, Krause T, Rocco A, Nolte CH, Villringer K, Fiebach JB. Spot sign in acute intracerebral hemorrhage in dynamic T1-weighted magnetic resonance imaging. *Stroke* 2016; 47: 417-423. Epub Dec 29, 2015.
14. Steiner I, Gomori JM, Melamed E. The prognostic value of the CT scan in conservatively treated patients with intracerebral hematoma. Stroke 1984; 15: 279-282.
15. Szepesi R, Szell IK, Hortobagyi T, Kardos L, Nagy K, Lanczi LI, Berenyi E, Bereczki D, Csiba L. New prognostic score for the prediction of 30-day outcome in spontaneous supratentorial cerebral haemorrhage. *Biomed Res Int* 2015; 2015: 961085
16. Toyoda K, Yasaka M, Nagata K, et al; Bleeding with Antithrombotic Therapy Study Group. Antithrombotic therapy influences location, enlargement, and mortality from intracerebral hemorrhage. The Bleeding with Antithrombotic Therapy (BAT) Retrospective Study. Cerebrovasc Dis 2009; 27: 151-159.
17. Yildiz OK, Arsava EM, Akpinar E, Topcuoglu MA. Previous antiplatelet use is associated with hematoma expansion in patients with spontaneous intracerebral hemorrhage. J Stroke Cerebrovasc Dis 2012; 21: 760-766.
18. Zazulia AR, Diringer MN, Derdeyn CP, Powers WJ. Progression of mass effect after intracerebral hemorrhage. Stroke 1999; 30: 1167-1173.

ii. Cohort of 10 patients or less

19. Bermejo PG, Garcia JA, Perez-Fernandez S, Arenillas JF. Spot sign and live-imaged dramatic intracerebral hematoma expansion. *Neurology* 2010; 75: 834.
20. Broderick JP, Brott TG, Tomsick T, Barsan W, Spilker J. Ultra-early evaluation of intracerebral hemorrhage. J Neurosurg 1990; 72: 195-199.
21. Chakraborty S, Blacquiere D, Lum C, Stotts G. Dynamic nature of the CT angiographic “spot sign”. *Br J Radiol* 2010; 83: e216–219.
22. Chakraborty S, Stotts G, Rush C, Hogan MJ, Dowlatshahi D. Dynamic 'spot sign' resolution following INR correction in a patient with warfarin-associated intracerebral hemorrhage. Case Rep Neurol 2011; 3: 154-159.
23. Chen ST, Chen SD, Hsu CY, Hogan EL. Progression of hypertensive intracerebral hemorrhage. Neurology 1989; 39: 1509-1514.
24. Freeman WD, Brott TG, Barrett KM, et al. Recombinant factor VIIa for rapid reversal of warfarin anticoagulation in acute intracranial hemorrhage. Mayo Clin Proc 2004; 79: 1495-1500.

25. Kelley RE, Berger JR, Scheinberg P, Stokes N. Active bleeding in hypertensive intracerebral hemorrhage: computed tomography. Neurology 1982; 32: 852-856.

iii. All patients had intracerebral haemorrhage secondary to an underlying structural cause identified on brain imaging

26. Delgado Almandoz JE, Kelly HR, Schaefer PW, et al. CT angiography spot sign predicts in-hospital mortality in patients with secondary intracerebral hemorrhage. J Neurointervent Surg 2012; 4: 442-447.
27. Kobata H, Sugie A, Yoritsune E, Miyata T, Toho T. Intracranial extravasation of contrast medium during diagnostic CT angiography in the initial evaluation of subarachnoid hemorrhage: report of 16 cases and review of the literature. *SpringerPlus* 2013; 2: 413.
28. Letourneau-Guillon L, Huynh T, Jakobovic R, Milwid R, Symons SP, Aviv RI. Traumatic intracranial hematomas: prognostic value of contrast extravasation. *AJNR Am J Neuroradiol* 2013; 34: 773-779.
29. van Asch CJ, Oudendijk JF, Rinkel GJ, Klijn CJ. Early intracerebral hematoma expansion after aneurysmal rupture. Stroke 2010; 41: 2592-2595.

iv. All patients received an intervention that might restrict ICH growth

30. Hallevi H, Gonzales NR, Barreto AD, et al. The effect of activated factor VII for intracerebral hemorrhage beyond 3 hours versus within 3 hours. Stroke 2008; 39: 473-475.
31. Inamasu J, Oheda M, Hayashi T, Kato Y, Hirose Y. Are admission systolic blood pressures predictive of outcomes in patients with spontaneous intracerebral haemorrhage after aggressive blood pressure management? *Eur J Emerg Med* 2015; 22: 170-175.
32. Kobayashi J, Koga M, Tanaka E, Okada Y, Kimura K, Yamagami H, Okuda S, Hasegawa Y, Shiokawa Y, Furui E, Nakagawara J, Kario K, Okata T, Arihiro S, Sato S, Nagatsuka K, Minematsu K, Toyoda K; SAMURAI Study Investigators. Continuous antihypertensive therapy throughout the initial 24 hours of intracerebral hemorrhage: the Stroke Acute Management with Urgent Risk-factor Assessment and Improvement-IntraCerebral Hemorrhage study. *Stroke* 2014; 45: 868-870.
33. Koga M, Arihiro S, Hasegawa Y, Shiokawa Y, Okada Y, Kimura K, Furui E, Nakagawara J, Yamagami H, Kario K, Okuda S, Tokunaga K, Takizawa H, Takasugi J, Sato S, Nagatsuka K, Minematsu K, Toyoda K; Stroke Acute Management with Urgent Risk-factor Assessment and Improvement Study Investigators. Intravenous nicardipine dosing for blood pressure lowering in acute intracerebral hemorrhage: the Stroke Acute Management with Urgent Risk-factor Assessment and Improvement-IntraCerebral Hemorrhage study. *J Stroke Cerebrovasc Dis* 2014; 23: 2780-2787.
34. Koga M, Toyoda K, Yamagami H, Okuda S, Okada Y, Kimura K, Shiokawa Y, Nakagawara J, Furui E, Hasegawa Y, Kario K, Osaki M, Miyagi T, Endo K, Nagatsuka K, Minematsu K; Stroke Acute Management with Urgent Risk-factor Assessment and Improvement Study Investigators. Systolic blood pressure lowering to 160 mmHg or less using nicardipine in acute intracerebral hemorrhage: a prospective, multicenter, observational study (the Stroke Acute Management with Urgent Risk-factor Assessment and Improvement-IntraCerebral Hemorrhage study). *J Hypertens* 2012; 30: 2357-2364.
35. Qureshi AI, Palesch YY, Martin R, et al; Antihypertensive Treatment of Acute Cerebral Hemorrhage Study Investigators. Effect of systolic blood pressure reduction on hematoma expansion, perihematomal edema, and 3-month outcome among patients with intracerebral hemorrhage: results from the antihypertensive treatment of acute cerebral hemorrhage study. Arch Neurol 2010; 67: 570-576.
36. Qureshi AI, Palesch YY, Martin R, et al; ATACH Investigators. Association of serum glucose concentrations during acute hospitalization with hematoma expansion, perihematomal edema, and three month outcome among patients with intracerebral hemorrhage. Neurocrit Care 2011; 15: 428-435.
37. Sakamoto Y, Koga M, Todo K, Okuda S, Okada Y, Kimura K, Shiokawa Y, Kamiyama K, Furui E, Hasegawa Y, Kario K, Okata T, Kobayashi J, Tanaka E, Yamagami H, Nagatsuka K, Minematsu K, Toyoda K; SAMURAI study investigators. Relative systolic blood pressure reduction and clinical outcomes in hyperacute intracerebral hemorrhage: the SAMURAI-ICH observational study. *J Hypertens* 2015; 33: 1069-1073.
38. Sakamoto Y; Koga M; Yamagami H; Okuda S; Okada Y; Kimura K; Shiokawa Y; Nakagawara J; Furui E; Hasegawa Y; Kario K; Arihiro S; Sato S; Kobayashi J; Tanaka E; Nagatsuka K; Minematsu K; Toyoda K; SAMURAI Study Investigators. Systolic blood pressure after intravenous antihypertensive treatment and clinical outcomes in hyperacute intracerebral hemorrhage: the Stroke Acute Management with Urgent Risk-factor Assessment and Improvement-IntraCerebral Hemorrhage study. *Stroke* 2013; 44: 1846-1851.
39. Sorimachi T, Fujii Y, Morita K, Tanaka R. Predictors of hematoma enlargement in patients with intracerebral hemorrhage treated with rapid administration of antifibrinolytic agents and strict blood pressure control. J Neurosurg 2007; 106: 250-254.
40. Tanaka E, Koga M, Kobayashi J, Kario K, Kamiyama K, Furui E, Shiokawa Y, Hasegawa Y, Okuda S, Todo K, Kimura K, Okada Y, Okata T, Arihiro S, Sato S, Yamagami H, Nagatsuka K, Minematsu K, Toyoda K. Blood pressure variability on antihypertensive therapy in acute intracerebral hemorrhage: the Stroke Acute Management with Urgent Risk-factor Assessment and Improvement-IntraCerebral Hemorrhage study. *Stroke* 2014; 45: 2275-2279.

41. Vespa P, McArthur D, Miller C, et al. Frameless stereotactic aspiration and thrombolysis of deep intracerebral hemorrhage is associated with reduction of hemorrhage volume and neurological improvement. Neurocrit Care 2005; 2: 274-281.

v. Overlap with another report of the same cohort

42. Appelboom G, Piazza M, Han JE, Bruce SS, Hwang B, Monahan A, Hwang RY, Kisslev S, Mayer S, Meyers PM, Badjatia N, Connolly ES Jr. von Willebrand factor genetic variant associated with hematoma expansion after intracerebral hemorrhage. *J Stroke Cerebrovasc Dis* 2013; 22: 713-717.
43. Barras CD, Tress BM, Christensen S, et al; Recombinant Activated Factor VII Intracerebral Hemorrhage Trial Investigators. Density and shape as CT predictors of intracerebral hemorrhage growth. Stroke 2009; 40: 1325-1331.
44. Barras CD, Tress BM, Christensen, Collins SM, Desmond PM, Skolnick BE, Mayer SA, Davis SM; Recombinant Activated Factor VII Intracerebral Hemorrhage Trial Investigators. Quantitative CT densitometry for predicting intracerebral hemorrhage growth. *AJNR Am J Neuroradiol* 2013; 34: 1139-1144.
45. Blacquiere D, Demchuk AM, Al-Hazzaa M, Deshpande A, Petrcich W, Aviv RI, Rodriguez-Luna D, Molina CA, Silva Blas Y, Dzialowski I, Czlonkowska A, Boulanger JM, Lum C, Gubitz G, Padma V, Roy J, Kase CS, Bhatia R, Hill MD, Dowlatshahi D; PREDICT/Sunnybrook ICH CTA Study Group. Intracerebral hematoma morphologic appearance on noncontrast computed tomography predicts significant hematoma expansion. *Stroke* 2015; 46: 3111-3116
46. Boulouis G, Dumas A, Betensky RA, Brouwers HB, Fotiadis P, Vashkevich A, Ayres A, Schwab K, Romero JM, Smith EE, Viswanathan A, Goldstein JN, Rosand J, Gurol ME, Greenberg SM. Anatomic pattern of intracerebral hemorrhage expansion: relation to CT angiography spot sign and hematoma center. *Stroke* 2014; 45: 1154-1156.
47. Broderick JP, Diringer MN, Hill MD, et al; Recombinant Activated Factor VII Intracerebral Hemorrhage Trial Investigators. Determinants of intracerebral hemorrhage growth: an exploratory analysis. Stroke 2007; 38: 1072-1075.
48. Brouwers HB, Biffi A, Ayres AM, et al. Apolipoprotein E genotype predicts hematoma expansion in lobar intracerebral hemorrhage. Stroke 2012; 43: 1490-1495.
49. Brouwers HB, Chang Y, Falcone GJ, Cai X, Ayres AM, Battey TW, Vashkevich A, McNamara KA, Valant V, Schwab K, Orzell SC, Bresette LM, Feske SK, Rost NS, Romero JM, Viswanathan A, Chou SH, Greenberg SM, Rosand J, Goldstein JN. Predicting hematoma expansion after primary intracerebral hemorrhage. *JAMA Neurol* 2014; 71: 158-164.
50. Brouwers HB, Falcone GJ, McNamara KA, et al. CTA spot sign predicts hematoma expansion in patients with delayed presentation after intracerebral hemorrhage. Neurocrit Care 2012; 17: 421-428.
51. Ciura VA, Brouwers HB, Pizzolato R, Ortiz CJ, Rosand J, Goldstein JN, Greenberg SM, Pomerantz SR, Gonzalez RG, Romero JM. Spot sign on 90-second delayed computed tomography angiography improves sensitivity for hematoma expansion and mortality: prospective study. *Stroke* 2014; 45: 3293-3297.
52. Cucchiara B, Messe S, Sansing L, Kasner S, Lyden P; CHANT Investigators. Hematoma growth in oral anticoagulant related intracerebral hemorrhage. Stroke 2008; 39: 2993-2996.
53. Davis SM, Broderick J, Hennerici M, et al; Recombinant Activated Factor VII Intracerebral Hemorrhage Trial Investigators. Hematoma growth is a determinant of mortality and poor outcome after intracerebral hemorrhage. Neurology 2006; 66: 1175-1181.
54. Delgado Almandoz JE, Yoo AJ, Stone MJ, et al. Systematic characterization of the computed tomography angiography spot sign in primary intracerebral hemorrhage identifies patients at highest risk for hematoma expansion: the spot sign score. Stroke 2009; 40: 2994-3000.
55. Delgado P, Cuadrado E, Rosell A, et al. Fas system activation in perihematomal areas after spontaneous intracerebral hemorrhage. Stroke 2008; 39: 1730-1734.
56. Evans A, Demchuk A, Symons SP, Dowlatshahi D, Gladstone DJ, Zhang L, Fox AJ, Aviv RI. The spot sign is more common in the absence of multiple prior microbleeds. *Stroke* 2010; 41: 2210-2217.
57. Flibotte JJ, Hagan N, O'Donnell J, Greenberg SM, Rosand J. Warfarin, hematoma expansion, and outcome of intracerebral hemorrhage. Neurology 2004; 63: 1059-1064.
58. Fujii Y, Tanaka R, Takeuchi S, Koike T, Minakawa T, Sasaki O. Hematoma enlargement in spontaneous intracerebral hemorrhage. J Neurosurg 1994; 80: 51-57.
59. Gazzola S, Aviv RI, Gladstone DJ, Mallia G, Li V, Fox AJ, Symons SP. Vascular and nonvascular mimics of the CT angiography "spot sign" in patients with secondary intracerebral hemorrhage. *Stroke* 2008; 39: 1177-1183.
60. Goldstein JN, Fazen LE, Snider R, et al. Contrast extravasation on CT angiography predicts hematoma expansion in intracerebral hemorrhage. Neurology 2007; 68: 889-894.
61. Huynh TJ, Aviv RI, Dowlatshahi D, Gladstone DJ, Laupacis A, Kiss A, Hill MD, Molina CA, Rodriguez-Luna D, Dzialowski I, Silva Y, Kobayashi A, Lum C, Boulanger JM, Gubitz G, Bhatia R, Padma V, Roy J, Kase CS, Symons SP, Demchuk AM; PREDICT/Sunnybrook CTA Investigators. Validation of the 9-point and 24-point hematoma expansion prediction scores and derivation of the PREDICT A/B scores. *Stroke* 2015; 46: 3105-3110.
62. Huynh TJ, Demchuk AM, Dowlatshahi D, Gladstone DJ, Krischek O, Kiss A, Hill MD, Molina CA, Rodriguez-Luna D, Dzialowski I, Silva Y, Czlonkowska A, Lum C, Boulanger JM, Gubitz G, Bhatia R, Padma V, Roy J, Kase

CS, Aviv RI; PREDICT/Sunnybrook ICH CTA Study Group. Spot sign number is the most important spot sign characteristic for predicting hematoma expansion using first-pass computed tomography angiography: analysis from the PREDICT study. *Stroke* 2013; 44: 972-977.
63. Jauch EC, Lindsell CJ, Adeoye O, et al. Lack of evidence for an association between hemodynamic variables and hematoma growth in spontaneous intracerebral hemorrhage. Stroke 2006; 37: 2061-2065.
64. Kazui S, Naritomi H, Yamamoto H, Sawada T, Yamaguchi T. Enlargement of spontaneous intracerebral hemorrhage. Incidence and time course. Stroke 1996; 27: 1783-1787.
65. Koculym A, Huynh TJ, Jakubovic R, Zhang L, Aviv RI. CT perfusion spot sign improves sensitivity for prediction of outcome compared with CTA and postcontrast CT. *AJNR Am J Neuroradiol* 2013; 34: 965-970.
66. Kuramatsu JB, Mauer C, Kiphuth IC, Lucking H, Kloska SP, Kohrmann M, Huttner HB. Reported antiplatelet use influences long-term outcome independently in deep intracerebral hemorrhage. *Neurosurgery* 2012; 70: 342-350.
67. Maas MB, Nemeth AJ, Rosenberg NF, Kosteva AR, Prabhakaran S, Naidech AM. Delayed intraventricular hemorrhage is common and worsens outcomes in intracerebral hemorrhage. Neurology 2013; 80: 1295-1299.
68. McCourt R, Gould B, Gioia L, Kate M, Coutts SB, Dowlatshahi D, Asdaghi N, Jeerakathil T, Hill MD, Demchuk AM, Buck B, Emery D, Butcher K; ICH ADAPT Investigators. Cerebral perfusion and blood pressure do not affect perihematoma edema growth in acute intracerebral hemorrhage. *Stroke* 2014; 45: 1292-1298.
69. McCourt R, Gould B, Kate M, Asdaghi N, Kosior JC, Coutts S, Hill MD, Demchuk A, Jeerakathil T, Emery D, Butcher KS. Blood-brain barrier compromise does not predict perihematoma edema growth in intracerebral hemorrhage. *Stroke* 2015; 46: 954-960.
70. Moussouttas M, Malhotra R, Fernandez L, et al. Impact of intraventricular hemorrhage upon intracerebral hematoma expansion. Neurocrit Care 2011; 14: 50-54.
71. Priglinger M, Arima H, Anderson C, Krause M; INTERACT Investigators. No relationship of lipid-lowering agents to hematoma growth: pooled analysis of the intensive blood pressure reduction in acute cerebral hemorrhage trials studies. *Stroke* 2015; 46: 857-859.
72. Radmanesh F, Falcone GJ, Anderson CD, Battey TW, Ayres AM, Vashkevich A, McNamara KA, Schwab K, Romero JM, Viswanathan A, Greenberg SM, Goldstein JN, Rosand J, Brouwers HB. Risk factors for computed tomography angiography spot sign in deep and lobar intracerebral hemorrhage are shared. *Stroke* 2014; 45: 1833-1835.
73. Rodriguez-Luna D, Dowlatshahi D, Aviv RI, Molina CA, Silva Y, Dzialowski I, Lum C, Czlonkowska A, Boulanger JM, K CS, Gubitz G, Bhatia R, Padma V, Roy J, Stewart T, Huynh TJ, Hill MD, Demchuk AM; PREDICT/Sunnybrook ICH CTA Study Group. Venous phase of computed tomography angiography increases spot sign detection, but intracerebral hemorrhage expansion is greater in spot signs detected in arterial phase. *Stroke* 2014; 45: 734-739.
74. Rodriguez-Luna D, Muchada M, Piñeiro S, Flores A, Rubiera M, Pagola J, Coscojuela P, Meler P, Sanjuan E, Boned-Riera S, Carcamo DA, Tomasello A, Alvarez-Sabin J, Ribo M, Molina CA. Potential blood pressure thresholds and outcome in acute intracerebral hemorrhage. *Eur Neurol* 2014; 72: 203-208.
75. Rodriguez-Luna D, Piñeiro S, Rubiera M, Ribo M, Coscojuela P, Pagola J, Flores A, Muchada M, Ibarra B, Meler P, Sanjuan E, Hernandez-Guillamon M, Alvarez-Sabin J, Montaner J, Molina CA. Impact of blood pressure changes and course on hematoma growth in acute intracerebral hemorrhage. *Eur J Neurol* 2013; 20: 1277-1283.
76. Rodriguez-Luna D, Rubiera M, Ribo M, Coscojuela P, Piñeiro S, Pagola J, Hernandez-Guillamon M, Ibarra B, Romero F, Alvarez-Sabin J, Montaner J, Molina CA. Ultraearly hematoma growth predicts poor outcome after acute intracerebral hemorrhage. *Neurology* 2011; 77: 1599-1604.
77. Rodriguez-Luna D, Stewart T, Dowlatshahi D, Kosior JC, Aviv RI, Molina CA, Silva Y, Dzialowski I, Lum C, Czlonkowska A, Boulanger JM, Kase CS, Gubitz G, Bhatia R, Padma V, Roy J, Subramaniam S, Hill MD, Demchuk AM; PREDICT/Sunnybrook ICH CTA Study Group. Perihematomal edema is greater in the presence of a spot sign but does not predict intracerebral hematoma expansion. *Stroke* 2016; 47: 350-355. Epub Dec 22, 2015.
78. Romero JM, Brouwers HB, Lu J, et al. Prospective validation of the computed tomographic angiography spot sign score for intracerebral hemorrhage. Stroke 2013; 44: 3097-3102.
79. Romero JM, Heit JJ, Delgado Almandoz JE, et al. Spot sign score predicts rapid bleeding in spontaneous intracerebral hemorrhage. Emerg Radiol 2012; 19: 195-202.
80. Sansing LH, Messe SR, Cucchiara BL, Cohen SN, Lyden PD, Kasner SE; CHANT Investigators. Prior antiplatelet use does not affect hemorrhage growth or outcome after ICH. Neurology 2009; 72: 1397- 1402.
81. Sato S, Arima H, Hirakawa Y, Heeley E, Delcourt C, Beer R, Li Y, Zhang J, Jüettler E, Wang J, Lavados PM, Robinson T, Lindley RI, Chalmers J, Anderson CS; INTERACT Investigators. The speed of ultraearly hematoma growth in acute intracerebral hemorrhage. *Neurology* 2014; 83: 2232-2238.
82. Silva Y, Leira R, Tejada J, Lainez JM, Castillo J, Dávalos A; Stroke Project, Cerebrovascular Diseases Group of the Spanish Neurological Society. Molecular signatures of vascular injury are associated with early growth of intracerebral hemorrhage. Stroke 2005; 36: 86-91.
83. Sorimachi T, Osada T, Baba T, Inoue G, Atsumi H, Ishizaka H, Hotta K, Hayashi N, Matsumae M. The striate artery, hematoma, and spot sign on coronal images of computed tomography angiography in putaminal intracerebral hemorrhage. *Stroke* 2013; 44: 1830-1832.
84. Staykov D, Wagner I, Volbers B, Hauer EM, Dörfler A, Schwab S, Bardutzky J. Natural course of perihemorrhagic edema after intracerebral hemorrhage. *Stroke* 2011; 42: 2625-2629.

85. Urday S, Beslow LA, Dai F, Zhang F, Battey TW, Vashkevich A, Ayres AM, Leasure AC, Selim MH, Simard JM, Rosand J, Kimberly WT, Sheth KN. Rate of perihematomal edema expansion predicts outcome after intracerebral hemorrhage. *Crit Care Med* 2016; 44: 790-797.
86. Urday S, Beslow LA, Goldstein DW, Vashkevich A, Ayres AM, Battey TW, Selim MH, Kimberly WT, Rosand J, Sheth KN. Measurement of perihematomal edema in intracerebral hemorrhage. *Stroke* 2015; 46: 1116-1119.
87. Wada R, Aviv RI, Fox AJ, et al. CT angiography "spot sign" predicts hematoma expansion in acute intracerebral hemorrhage. Stroke 2007; 38: 1257-1262.
88. Wagner I, Hauer EM, Staykov D, Volbers B, Dörfler A, Schwab S, Bardutzky J. Effects of continuous hypertonic saline infusion on perihemorrhagic edema evolution. *Stroke* 2011; 42: 1540-1545.
89. Wang X, Arima H, Al-Shahi Salman R, Woodward M, Heeley E, Stapf C, Lavados PM, Robinson T, Huang Y, Wang J, Delcourt C, Anderson CS; INTERACT Investigators. Clinical prediction algorithm (BRAIN) to determine risk of hematoma growth in acute intracerebral hemorrhage. *Stroke* 2015; 46: 376-381.
90. Yang J; Arima H; Wu G; Heeley E Delcourt C; Zhou J; Chen G; Wang X; Zhang S; Yu S; Chalmers J; Anderson CS; INTERACT Investigators. Prognostic significance of perihematomal edema in acute intracerebral hemorrhage: pooled analysis from the intensive blood pressure reduction in acute cerebral hemorrhage trial studies. *Stroke* 2015; 46: 1009-1013.

B. Cohorts eligible for inclusion invited to share individual patient data, but did not provide data

i. Authors agreed initially, but did not respond

1. Diedler J, Sykora M, Rupp A, et al. Impaired cerebral vasomotor activity in spontaneous intracerebral hemorrhage. Stroke 2009; 40: 815-819.
2. Du Q, Yang DB, Shen YF, Yu WH, Zhang ZY, Zhu Q, Che ZH, Liu QJ, Wang H, Dong XQ. Plasma leptin level predicts hematoma growth and early neurological deterioration after acute intracerebral hemorrhage. *Peptides* 2013; 45: 35-39.
3. Ducruet AF, Hickman ZL, Zacharia BE, et al. Impact of platelet transfusion on hematoma expansion in patients receiving antiplatelet agents before intracerebral hemorrhage. Neurol Res 2010; 32: 706-710.
4. Fujitsu K, Muramoto M, Ikeda Y, Inada Y, Kim I, Kuwabara T. Indications for surgical treatment of putaminal hemorrhage. Comparative study based on serial CT and time-course analysis. J Neurosurg 1990; 73: 518-525.
5. Hagii J, Tomita H, Metoki N, Saito S, Shiroto H, Hitomi H, Kamada T, Seino S, Takahashi K, Baba Y, Sasaki S, Uchizawa T, Iwata M, Matsumoto S, Osanai T, Yasujima M, Okumura K. Characteristics of intracerebral hemorrhage during rivaroxaban treatment: comparison with those during warfarin. *Stroke* 2014; 45: 2805-2807.
6. Hallevi H, Abraham AT, Barreto AD, Grotta JC, Savitz SI. The spot sign in intracerebral hemorrhage: the importance of looking for contrast extravasation. Cerebrovasc Dis 2010; 29: 217-220.
7. Han JH, Lee JM, Koh EJ, Choi HY. The spot sign predicts hematoma expansion, outcome, and mortality in patients with primary intracerebral hemorrhage. *J Korean Neurosurg Soc* 2014; 56: 303-309.
8. Hotta K, Sorimachi T, Osada T, Baba T, Inoue G, Atsumi H, Ishizaka H, Matsuda M, Hayashi N, Matsumae M. Risks and benefits of CT angiography in spontaneous intracerebral hemorrhage. *Acta Neurochir (Wien)* 2014; 156: 911-917.
9. Hou XY, Gao PY. Perihematomal perfusion typing and spot sign of acute intracerebral hemorrhage with multimode computed tomography: a preliminary study. *Chin Med Sci J* 2014; 29: 139-143.
10. Ji N, Lu JJ, Zhao YL, Wang S, Zhao JZ. Imaging and clinical prognostic indicators for early hematoma enlargement after spontaneous intracerebral hemorrhage. Neurol Res 2009; 31: 362-366.
11. Kim SH, Jung HH, Whang K, Kim JY, Pyen JS, Oh JW. Which emphasizing factors are most predictive of hematoma expansion in spot sign positive intracerebral hemorrhage? *J Korean Neurosurg Soc* 2014; 56: 86-90.
12. Li N, Liu YF, Ma L, Worthmann H, Wang YL, Wang YJ, Gao YP, Raab P, Dengler R, Weissenborn K, Zhao XQ. Association of molecular markers with perihematomal edema and clinical outcome in intracerebral hemorrhage. *Stroke* 2013; 44: 658-663.
13. Li N, Wang Y, Wang W, et al. Contrast extravasation on computed tomography angiography predicts clinical outcome in primary intracerebral hemorrhage: a prospective study of 139 cases. Stroke 2011; 42: 3441-3446.
14. Lim JK, Hwang HS, Cho BM, et al. Multivariate analysis of risk factors of hematoma expansion in spontaneous intracerebral hemorrhage. Surg Neurol 2008; 69: 40-45.
15. Liu SF, Li YW, Wang X, Xu XL. Relationship of cerebral microbleeds with hematoma growth in elderly patients with acute hypertensive intracerebral hemorrhage. *Chin J Contemp Neurol Neurosurg* 2015; 15: 53-56.
16. Mould WA, Carhuapoma JR, Muschelli J, Lane K, Morgan TC, McBee NA, Bistran-Hall AJ, Ullman NL, Vespa P, Martin NA, Awad I, Zuccarello M, Hanley DF; MISTIE Investigators. Minimally invasive surgery plus recombinant tissue-type plasminogen activator for intracerebral hemorrhage evacuation decreases perihematomal edema. *Stroke* 2013; 44: 627-634.
17. Moussouttas M, Malhotra R, Fernandez L, et al. Role of antiplatelet agents in hematoma expansion during the acute period of intracerebral hemorrhage. Neurocrit Care 2010; 12: 24-29.
18. Okada T, Nakase T, Sasaki M, Ishikawa T. Do the antithrombotic therapy at the time of intracerebral hemorrhage influence clinical outcome? Analysis between the difference of antiplatelet and anticoagulant agents and clinical

course. *J Stroke Cerebrovasc Dis* 2014; 23: 1781-1788.
19. Ovesen C, Christensen AF, Krieger DW, Rosenbaum S, Havsteen I, Christensen H. Time course of early postadmission hematoma expansion in spontaneous intracerebral hemorrhage. *Stroke* 2014; 45: 994-999.
20. Park SY, Kong MH, Kim JH, Kang DS, Song KY, Huh SK. Role of 'spot sign' on CT angiography to predict hematoma expansion in spontaneous intracerebral hemorrhage. J Korean Neurosurg Soc 2010; 48: 399-405.
21. Rizos T, Dorner N, Jenetzky E, Sykora M, Mundiyanapurath S, Horstmann S, Veltkamp R, Rohde S, Bendszus M, Steiner T. Spot signs in intracerebral hemorrhage: useful for identifying patients at risk for hematoma enlargement? *Cerebrovasc Dis* 2013; 35: 582-589.
22. Rosa Junior M, da Rocha AJ, Saade N, Maia Junior ACM, Gagliardi RJ. Active extravasation of contrast within the hemorrhage (spot sign): a multidetector computed tomography finding that predicts growth and a worse prognosis in non-traumatic intracerebral hemorrhage. *Arq Neuropsiquiatr* 2013; 71: 791-797.
23. Su M, Jiang Y, Huang GY, Hu SH, Deng MH. Clinical observation of hemocoagulase combined with aminomethylbenzoic acid in the treatment of basal ganglia hemorrhage. *Chin J Contemp Neurol Neurosurg* 2014; 14: 625-627.
24. Sun SJ, Gao PY, Sui BB, Hou XY, Lin Y, Xue J, Zhai RY. "Dynamic spot sign" on CT perfusion source images predicts haematoma expansion in acute intracerebral haemorrhage. *Eur Radiol* 2013; 23: 1846-1854.
25. Takeda R, Ogura T, Ooigawa H, Fushihara G, Yoshikawa S, Okada D, Araki R, Kurita H. A practical prediction model for early hematoma expansion in spontaneous deep ganglionic intracerebral hemorrhage. *Clin Neurol Neurosurg* 2013; 115: 1028-1031.
26. Teernstra OP, Evers SM, Lodder J, Leffers P, Franke CL, Blaauw G. Stereotactic treatment of intracerebral hematoma by means of a plasminogen activator: a multicenter randomized controlled trial (SICHPA). Stroke 2003; 34: 968-974.
27. Tsukabe A, Watanabe Y, Tanaka H, Kunitomi Y, Nishizawa M, Arisawa A, Yoshiya K, Shimazu T, Tomiyama N. Prevalence and diagnostic performance of computed tomography angiography spot sign for intracerebral hematoma expansion depend on scan timing. *Neuroradiology* 2014; 56: 1039-1045.
28. Wang YH, Fan JY, Luo GD, et al. Hematoma volume affects the accuracy of computed tomographic angiography 'spot sign' in predicting hematoma expansion after acute intracerebral hemorrhage. Eur Neurol 2011; 65: 150-155.
29. Yaghi S, Dibu J, Achi E, Patel A, Samant R, Hinduja A. Hematoma expansion in spontaneous intracerebral hemorrhage: predictors and outcome. *Int J Neurosci* 2014; 124: 890-893.
30. Yang NR, Kim SJ, Seo EK. Spontaneous intracerebral hemorrhage with antiplatelets/anticoagulants/none: a comparison analysis. *Acta Neurochir (Wien)* 2014; 156: 1319-1325.
31. Yu YL, Kumana CR, Lauder IJ, et al. Treatment of acute cerebral hemorrhage with intravenous glycerol. A double-blind, placebo-controlled, randomized trial. Stroke 1992; 23: 967-971.

ii. *Investigators declined to participate*

32. Huttner HB, Schellinger PD, Hartmann M, et al. Hematoma growth and outcome in treated neurocritical care patients with intracerebral hemorrhage related to oral anticoagulant therapy: comparison of acute treatment strategies using vitamin K, fresh frozen plasma, and prothrombin complex concentrates. Stroke 2006; 37: 1465-1470.
33. Kuwashiro T, Yasaka M, Itabashi R, et al. Enlargement of acute intracerebral hematomas in patients on long-term warfarin treatment. Cerebrovasc Dis 2010; 29: 446-453.
34. Naidech AM, Jovanovic B, Liebling S, et al. Reduced platelet activity is associated with early clot growth and worse 3-month outcome after intracerebral hemorrhage. Stroke 2009; 40: 2398-2401.
35. Ohwaki K, Yano E, Nagashima H, Hirata M, Nakagomi T, Tamura A. Blood pressure management in acute intracerebral hemorrhage: relationship between elevated blood pressure and hematoma enlargement. Stroke 2004; 35: 1364-1367.
36. Perez ES, Delgado-Mederos R, Rubiera M, et al. Transcranial duplex sonography for monitoring hyperacute intracerebral hemorrhage. Stroke 2009; 40: 987-990.
37. Yasaka M, Minematsu K, Naritomi H, Sakata T, Yamaguchi T. Predisposing factors for enlargement of intracerebral hemorrhage in patients treated with warfarin. Thromb Haemost 2003; 89: 278-283.
38. Zuccarello M, Brott T, Derex L, et al. Early surgical treatment for supratentorial intracerebral hemorrhage: a randomized feasibility study. Stroke 1999; 30: 1833-1839.

iii. Data sent were incomplete

39. Ederies A, Demchuk A, Chia T, et al. Postcontrast CT extravasation is associated with hematoma expansion in CTA spot negative patients. Stroke 2009; 40: 1672-1676.
40. Koch S, Romano JG, Forteza AM, Otero CM, Rabinstein AA. Rapid blood pressure reduction in acute intracerebral hemorrhage: feasibility and safety. Neurocrit Care 2008; 8: 316-321.
41. Zubkov AY, Mandrekar JN, Claassen DO, Manno EM, Wijdicks EF, Rabinstein AA. Predictors of outcome in warfarin-related intracerebral hemorrhage. Arch Neurol 2008; 65: 1320-1325.

Characteristics of included studies

Publication	Location(s)	Hospitals	Recruiting location	Study period	Study design
Abilleira (2003)	Spain	1	Any	12/99 - 03/01	Prospective
Alvarez-Sabin (2004)	Spain	1	Emergency department	09/01 - 05/02	Prospective
Anderson (2008)	Australia, China, South Korea	44	Any	11/05 - 04/07	Prospective
Anderson (2013)	Many countries	121	Any	10/08 - 08/12	Prospective
Bakhshayesh (2014)	Iran	1	Emergency department	02/11 - 07/12	Prospective
Biffi (2011)	USA	1	Emergency department	01/99 - 12/09	Prospective
Brott (1997)	USA	12	Emergency department	02/89 - 04/94	Prospective
Brouwers (2015)	USA	1	Emergency department	04/12 - 05/13	Prospective
Butcher (2013)	Canada	4	Any	01/07 - 02/12	Prospective
Delgado (2006)	Spain	1	Emergency department	03/01 - 03/04	Prospective
Di Napoli (2014)	Argentina, Italy, Romania	3	Any	01/09 - 12/11	Prospective
d’Esterre (2011)	Canada	1	Stroke unit	03/05 - 09/09	Prospective
Fujii (1998)	Japan	1	Neurosurgical unit	01/87 - 12/95	Retrospective
Kawano-Castillo (2014)	USA	1	Emergency department	11/09 - 04/13	Prospective
Kazui (1997)	Japan	1	Stroke unit	01/85 - 12/94	Retrospective
Leira (2004)	Spain	15	Emergency department	05/99 - 04/01	Prospective
Li (2013)	Germany	1	Any	12/09 - 06/12	Prospective
Li (2015)	China	1	Any	05/11 - 01/15	Retrospective
Marti-Fabregas (2010)	Spain	2	Any	05/05 - 05/09	Prospective
Moon (2014)	South Korea	1	Any	01/05 - 09/12	Retrospective
Murai (1999)	Japan	1	Any	11/97 - 12/98	Prospective
Rodriguez-Luna (2011)	Spain	1	Emergency department	04/09 - 06/10	Prospective
Rodriguez-Luna (2014)	Many countries	12	Any	06/06 - 02/12	Prospective
Toyoda (2005)	Japan	1	Stroke unit	01/99 - 02/05	Retrospective
Venkatasubramanian (2011)	USA	1	Any	08/04 - 06/08	Prospective
Volbers (2015)	Germany	1	Any	01/06 - 02/10	Retrospective
Witsch (2015)	USA	1	Neuro-critical care unit	02/09 - 03/14	Prospective
Yao (2015)	USA	1	Any	10/07 - 03/14	Retrospective
Zazulia (2009)	USA	1	Neuro-critical care unit	03/05 - 10/06	Retrospective
Zazulia (2011)	USA	1	Neuro-critical care unit	01/02 - 12/04	Retrospective
Ziai (2003)	USA	1	Neuro-critical care unit	09/96 - 08/99	Retrospective
VISTA-ICH (5 studies)	Many countries	Many	Any	08/01 - 02/07	Prospective

Numbers of patients provided by each study and number included in master dataset

Study	Patients with repeat assessment of volume	Not included in published study	Also included in another participating study	Not spontaneous primary intracerebral haemorrhage	Treatment affecting ICH volume	Follow-up scan(s) done after surgery	Patients included in master dataset
Abilleira (2003)	42	0	0	0	0	0	42
Alvarez-Sabin (2004)	19	0	0	0	0	0	19
Anderson (2008)	172	0	0	0	5	**?**	167
Anderson (2013)	473	0	0	0	7	**?**	466
Bakhshayesh (2014)	52	0	0	0	0	0	52
Biffi (2011)	843	0	**?**	33	**?**	0	810
Brott (1997)	103	0	0	0	0	0	103
Brouwers (2015)	97	0	0	0	0	0	97
Butcher (2013)	36	0	0	0	0	0	36
d'Esterre (2011)	14	0	0	0	**?**	**?**	14
Delgado (2006)	49	0	15	0	0	0	34
Di Napoli (2014)	394	0	0	0	0	0	394
Fujii (1998)	627	0	0	0	0	0	627
Kawano-Castillo (2014)	58	0	0	1	0	0	57
Kazui (1997)	191	**?**	0	0	0	**?**	191
Leira (2004)	250	0	0	0	0	2	248
Li (2013)	21	0	0	0	0	0	21
Li (2015)	172	0	0	0	0	0	172
Marti-Fabregas (2010)	90	0	**?**	0	0	0	90
Moon (2014)	251	0	0	0	0	**?**	251
Murai (1999)	24	0	0	0	0	**?**	24
Rodriguez-Luna (2011)	94	0	0	0	0	0	94
Rodriguez-Luna (2014) – PREDICT	318	0	57	0	0	0	261
Toyoda (2005)	204	14	0	0	0	**?**	190
Venkatasubramanian (2011)	27	0	0	0	0	0	27
Volbers (2015)	205	0	0	0	0	0	205
Witsch (2015)	357	132	0	5	0	**?**	220
Yao (2015)	237	0	0	0	0	0	237
Zazulia (2009)	25	12	3	0	0	0	10
Zazulia (2011)	16	1	0	0	0	0	15
Ziai (2003)	21	0	0	0	0	0	21
VISTA-ICH (5 studies)	946	0	-	19	-	7	920
**Total**	**6,428**	**159**	**75**	**58**	**12**	**9**	**6,115**

Epochs of each included cohort

Study	Start Date	End Date
Brott (1997)	16-Feb-1989	30-Apr-1994
Kazui (1997)	01-Jan-1985	31-Dec-1994
Fujii (1998)	Jan-1987	Dec-1995
Murai (1999)	Nov-1997	Dec-1998
Ziai (2003)	Sep-1996	Aug-1999
Abilleira (2003)	12-Dec-1999	26-Mar-2001
Leira (2004)	May-1999	Apr-2001
Alvarez-Sabin (2004)	27-Sep-2001	07-May-2002
Delgado (2006)	Mar-2001	Mar-2004
Zazulia (2011)	Jan-2002	Dec-2004
Toyoda (2005)	Jan-1999	Feb-2005
Zazulia (2009)	Mar-2005	Oct-2006
Anderson (2008) – INTERACT1	Nov-2005	Apr-2007
Venkatasubramanian (2011)	Aug-2004	Jun-2008
Marti-Fabregas (2010)	May-2005	May-2009
d'Esterre (2011)	15-Mar-2005	26-Sep-2009
Biffi (2011)	01-Jan-1999	31-Dec-2009
Volbers (2015)	Jan-2006	Feb-2010
Rodriguez-Luna (2011)	Apr-2009	Jun-2010
Butcher (2013) – ICH ADAPT	28-Jan-2007	06-Dec-2011
Di Napoli (2014)	01-Jan-2009	31-Dec-2011
Rodriguez-Luna (2014) - PREDICT	24-Jun-2006	27-Feb-2012
Li (2013)	Dec-2009	Jun-2012
Bakhshayesh (2014)	Feb-2011	Jul-2012
Anderson (2013) – INTERACT2	Oct-2008	Aug-2012
Moon (2014)	01-Jan-2005	15-Sep-2012
Kawano-Castillo (2014)	24-Nov-2009	22-Apr-2013
Brouwers (2015)	Apr-2012	May-2013
Yao (2015)	Oct-2007	Mar-2014
Witsch (2015)	Feb-2009	Mar-2014
Li (2015)	May-2011	Jan-2015
VISTA:		
Lyden (2007) – CHANT	Jul-2004	Sep-2005
Mayer (2005a) – F7ICH-1389	Aug-2001	Oct-2002
Mayer (2006) – F7ICH-2073	Nov-2001	Mar-2003
Mayer (2005b) – F7ICH-1371	Aug-2002	Mar-2004
Mayer (2008) – FAST	May-2005	Feb-2007

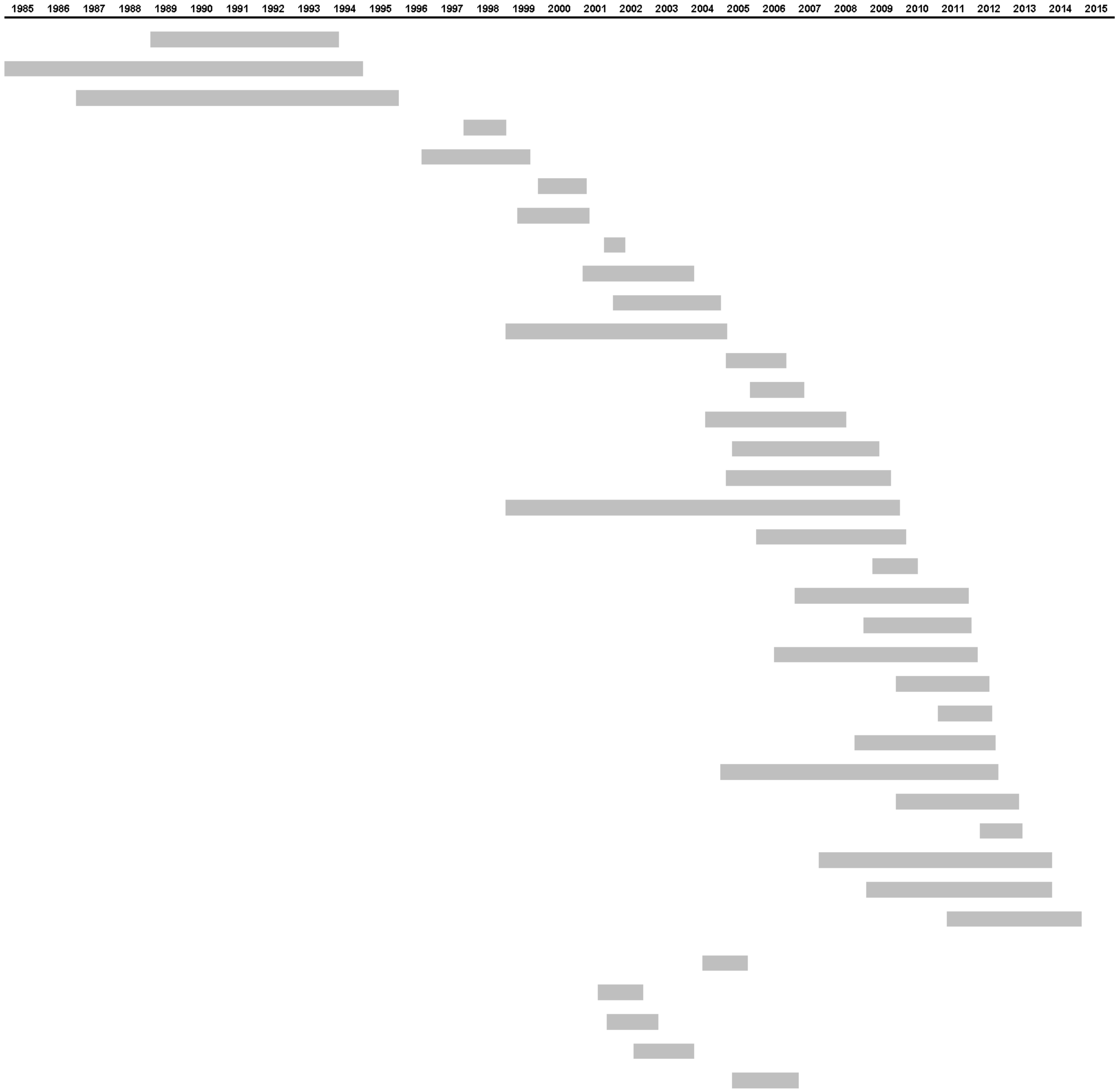

Numbers of patients in the master dataset who were included in the analysis dataset

Study	Records included in master dataset	Time from onset to baseline scan not known	Time from onset to baseline scan <0.5 hours	Time from onset to baseline scan >24 hours	Time from onset to follow-up scan not known	Time from onset to follow-up scan >6 days	ICH volume on baseline scan >150 ml	Records included in analysis dataset
Abilleira (2003)	42	8	0	0	0	0	0	34
Alvarez-Sabin (2004)	19	0	0	0	0	1	0	18
Anderson (2008)	167	1	7	0	0	0	0	159
Anderson (2013)	466	1	14	0	0	0	0	451
Bakhshayesh (2014)	52	0	0	0	0	0	0	52
Biffi (2011)	810	253	0	48	0	2	1	506
Brott (1997)	103	1	2	0	0	0	0	100
Brouwers (2015)	97	25	3	2	0	0	1	66
Butcher (2013)	36	0	0	0	0	0	0	36
d'Esterre (2011)	14	0	0	0	1	0	0	13
Delgado (2006)	34	2	0	0	0	6	0	26
Di Napoli (2014)	394	1	11	0	0	0	3	379
Fujii (1998)	627	0	30	0	0	0	2	595
Kawano-Castillo (2014)	57	2	0	0	3	0	1	51
Kazui (1997)	191	1	1	0	0	0	0	189
Leira (2004)	248	1	0	0	0	4	2	241
Li (2013)	21	0	0	0	0	0	0	21
Li (2015)	172	0	0	0	0	0	0	172
Marti-Fabregas (2010)	90	3	1	0	21	0	0	65
Moon (2014)	251	0	14	0	22	38	0	177
Murai (1999)	24	0	0	0	0	0	0	24
Rodriguez-Luna (2011)	94	0	0	0	0	2	0	92
Rodriguez-Luna (2014) – PREDICT	261	0	2	0	6	0	0	253
Toyoda (2005)	190	2	2	9	0	0	0	177
Venkatasubramanian (2011)	27	0	0	2	0	0	0	25
Volbers (2015)	205	0	0	5	0	0	0	200
Witsch (2015)	220	25	3	9	8	0	0	175
Yao (2015)	237	0	4	0	0	0	7	226
Zazulia (2009)	10	0	0	1	0	0	0	9
Zazulia (2011)	15	0	0	5	0	0	0	10
Ziai (2003)	21	5	0	2	0	0	0	14
VISTA-ICH	920	0	37	0	2	2	0	879
**Total**	**6,115**	**331**	**131**	**83**	**63**	**55**	**17**	**5,435**

Number of patients included in datasets used for analyses

Study	Patients not taking anticoagulant therapy	Patients taking anticoagulant therapy	Patients with use of anticoagulant therapy specified	Patients undergoing CTA with information on spot sign presence
Abilleira (2003)	34	0	0	0
Alvarez-Sabin (2004)	18	0	0	0
Anderson (2008) – INTERACT1	158	1	159	0
Anderson (2013) – INTERACT2	435	16	451	0
Bakhshayesh (2014) ¶	44	0	0	0
Biffi (2011)	403	103	506	273
Brott (1997)	100	0	0	0
Brouwers (2015)	56	10	66	66
Butcher (2013) – ICH ADAPT	32	4	36	0
d'Esterre (2011)	12	1	13	13
Delgado (2006) *	25	1	0	0
Di Napoli (2014)	351	28	379	0
Fujii (1998)	595	0	0	0
Kawano-Castillo (2014) †	50	1	0	0
Kazui (1997)	189	0	0	0
Leira (2004)	241	0	0	0
Li (2013)	20	1	21	0
Li (2015)	172	0	0	0
Marti-Fabregas (2010)	65	0	0	0
Moon (2014)	172	5	177	177
Murai (1999)	24	0	0	24
Rodriguez-Luna (2011)	92	0	0	64
Rodriguez-Luna (2014) – PREDICT	224	29	253	251
Toyoda (2005)	177	0	0	0
Venkatasubramanian (2011)	25	0	0	0
Volbers (2015)	171	29	200	0
Witsch (2015)	150	25	175	0
Yao (2015)	173	53	226	0
Zazulia (2009)	8	1	9	0
Zazulia (2011)	10	0	0	0
Ziai (2003)	14	0	0	0
VISTA-ICH ‡	836	43	879	0
**Total**	**5,076**	**351**	**3,550**	**868**

¶ Eight patients known to have been taking anticoagulant therapy and/or antiplatelet therapy at symptom onset but not known whether they were taking anticoagulant therapy only, antiplatelet therapy only, or both so they have not been included in any of the resulting datasets.
* Oral anticoagulant intake was an exclusion criterion. The patient with a 'Yes' for anticoagulant therapy at symptom onset was either receiving a non-oral anticoagulant or should not have been included in the study.
† Known coagulopathy, including use of anticoagulant therapy, was an exclusion criterion. The patient with a 'Yes' for anticoagulant therapy at symptom onset should not have been included in the study.
‡ Known use of oral anticoagulant therapy was an exclusion criterion in four trials, but not in a fifth trial.

Aggregation of studies into separate datasets for analysis

		**Not taking anticoagulant therapy**	**Taking anticoagulant therapy**	
**Cohort including patients taking anticoagulant therapy**	**No †**	1,875 patients<br>17 cohorts	2 patients<br>2 cohorts	1,877 patients<br>17 cohorts
	**Yes ‡**	3,201 patients<br>19 cohorts	349 patients<br>19 cohorts	**3,550 patients**<br>**19 cohorts**
		**5,076 patients**<br>**36 cohorts**	**351 patients**<br>**21 cohorts**	5,427 patients *<br>36 cohorts

† Cohort in which either taking anticoagulant therapy at symptom onset was an exclusion criterion or none of the patients included in the cohort were supposed to be taking anticoagulant therapy at symptom onset.
‡ Cohort in which taking anticoagulant therapy at symptom onset was not an exclusion criterion and at least one patient included in the cohort was taking anticoagulant therapy at symptom onset.
* There were eight patients in the 'analysis' dataset from the Bakhshayesh (2014) cohort known to have been taking anticoagulant therapy and/or antiplatelet therapy but it is not known whether this was anticoagulant therapy only, antiplatelet therapy only, or both so they have not been included in any of the resulting datasets.

Associations between each predictor and ICH growth >6ml in patients who were not taking anticoagulant therapy at symptom onset.
The adjusted odds ratio for each predictor is adjusted for both time from symptom onset to baseline scan and ICH volume on baseline scan.

			Unadjusted		Adjusted	
**Predictor**	**Number of patients (cohorts)**	**Comparison**	**Odds ratio (95% CI)**	**p-value**	**Odds ratio (95% CI)**	**p-value**
Sex	4,884 (35)	Male versus Female	1·23 (1·06 to 1·42)	0·007	1·26 (1·08 to 1·48)	0·004
Age (years)	5,074 (36)	76 versus 56	1·07 (0·96 to 1·20)	0·23	1·12 (0·99 to 1·26)	0·068
Previous stroke	4,471 (29)	Yes versus No	1·10 (0·89 to 1·37)	0·38	1·25 (0·98 to 1·58)	0·069
Previous ICH	2,542 (16)	Yes versus No	1·33 (0·91 to 1·93)	0·14	1·39 (0·92 to 2·12)	0·12
Previous ischaemic stroke	2,666 (21)	Yes versus No	1·10 (0·78 to 1·54)	0·58	1·30 (0·89 to 1·90)	0·18
History of hypertension	5,050 (36)	Yes versus No	0·77 (0·65 to 0·90)	0·001	0·85 (0·72 to 1·01)	0·071
History of diabetes mellitus	4,197 (31)	Yes versus No	0·98 (0·80 to 1·19)	0·81	1·01 (0·81 to 1·25)	0·96
History of liver disease	3,248 (19)	Yes versus No	1·75 (1·29 to 2·39)	0·0004	1·68 (1·21 to 2·34)	0·002
History of excessive alcohol consumption	3,029 (23)	Yes versus No	1·37 (1·08 to 1·72)	0·008	1·30 (1·02 to 1·66)	0·036
Antiplatelet therapy at symptom onset	4,170 (31)	Yes versus No	1·30 (1·07 to 1·57)	0·007	1·47 (1·20 to 1·81)	0·0003
Systolic blood pressure at presentation (mmHg)	4,882 (36)	198 versus 158	1·06 (0·96 to 1·16)	0·24	1·04 (0·94 to 1·15)	0·43
Blood glucose at presentation (mmol/l)	4,265 (36)	8·7 versus 5·9	1·14 (1·06 to 1·22)	0·0004	1·01 (0·93 to 1·09)	0·79
Platelet count at presentation (x10^9/l)	3,857 (36)	266 versus 181	0·79 (0·71 to 0·88)	<0·0001	0·79 (0·71 to 0·89)	<0·0001
Glasgow Coma Scale score at presentation	4,564 (32)	3 - 6 versus 15	3·25 (2·35 to 4·48)	<0·0001	1·32 (0·93 to 1·88)	0·055
		7 - 12 versus 15	2·83 (2·34 to 3·43)		1·29 (1·05 to 1·60)	
		13 - 14 versus 15	1·99 (1·64 to 2·40)		1·27 (1·03 to 1·56)	
NIHSS score at presentation	2,661 (35)	18 versus 7	3·33 (2·77 to 3·99)	<0·0001	1·72 (1·41 to 2·10)	<0·0001
Location of ICH on baseline scan	4,920 (35)	Lobar versus Non-lobar	1·69 (1·43 to 2·00)	<0·0001	1·08 (0·89 to 1·30)	0·46
IVH present on baseline scan	4,980 (36)	Yes versus No	1·13 (0·98 to 1·32)	0·096	0·82 (0·70 to 0·97)	0·019

Characteristics of patients who were not taking anticoagulant therapy at ICH onset, who were included in the development and validation of the model predicting ICH growth >6ml

Characteristic	Category or values	Development (n = 2,534)	Validation (n = 945)
Sex	Total	2,533	945
	Male	1,512 (60%)	560 (59%)
Age (years)	Total	2,534	945
	Median (lower & upper quartile)	68 (58, 77)	64 (54, 76)
Previous stroke	Total	2,520	926
	Yes	341 (14%)	114 (12%)
Previous ICH	Total	1,332	926
	Yes	75 (6%)	51 (6%)
Previous ischaemic stroke	Total	1,334	926
	Yes	121 (9%)	70 (8%)
History of hypertension	Total	2,532	943
	Yes	1,954 (77%)	651 (69%)
History of diabetes mellitus	Total	2,530	926
	Yes	429 (17%)	146 (16%)
History of liver disease	Total	1,493	548
	Yes	74 (5%)	34 (6%)
History of excessive alcohol consumption	Total	685	916
	Yes	143 (21%)	172 (19%)
Antiplatelet therapy at symptom onset	Total	2,534	945
	Yes	545 (22%)	223 (24%)
Systolic blood pressure at presentation (mmHg)	Total	2,520	942
	Median (lower & upper quartile)	178 (160, 197)	172 (150, 197)
Blood glucose at presentation (mmol/l)	Total	2,443	909
	Median (lower & upper quartile)	6·9 (5·9, 8·5)	7·0 (5·9, 8·8)
Platelet count at presentation ($x10^9$/l)	Total	1,532	939
	Median (lower & upper quartile)	224 (189, 271)	214 (170, 259)
Glasgow Coma Scale score at presentation	Total	2,523	925
	3 - 6	119 (5%)	81 (9%)
	7 - 12	572 (23%)	264 (29%)
	13 - 14	640 (25%)	180 (19%)
	15	1,192 (47%)	400 (43%)
NIHSS score at presentation	Total	1,726	394
	Median (lower & upper quartile)	13 (8, 17)	12 (6, 18)
Time from symptom onset to baseline scan (hours)	Total	2,534	945
	Median (lower & upper quartile)	2·0 (1·2, 3·4)	2·5 (1·2, 4·9)
ICH volume on baseline scan (mL)	Total	2,534	945
	Median (lower & upper quartile)	13 (7, 28)	14 (6, 31)
Location of ICH on baseline scan	Total	2,432	942
	Lobar	513 (21%)	275 (29%)
IVH present on baseline scan	Total	2,439	945
	Yes	849 (35%)	353 (37%)
ICH growth >6ml	Total	2,534	945
	Yes	560 (22%)	211 (22%)

Calibration of the prediction model in the validation dataset of patients who were not taking anticoagulant therapy at ICH onset

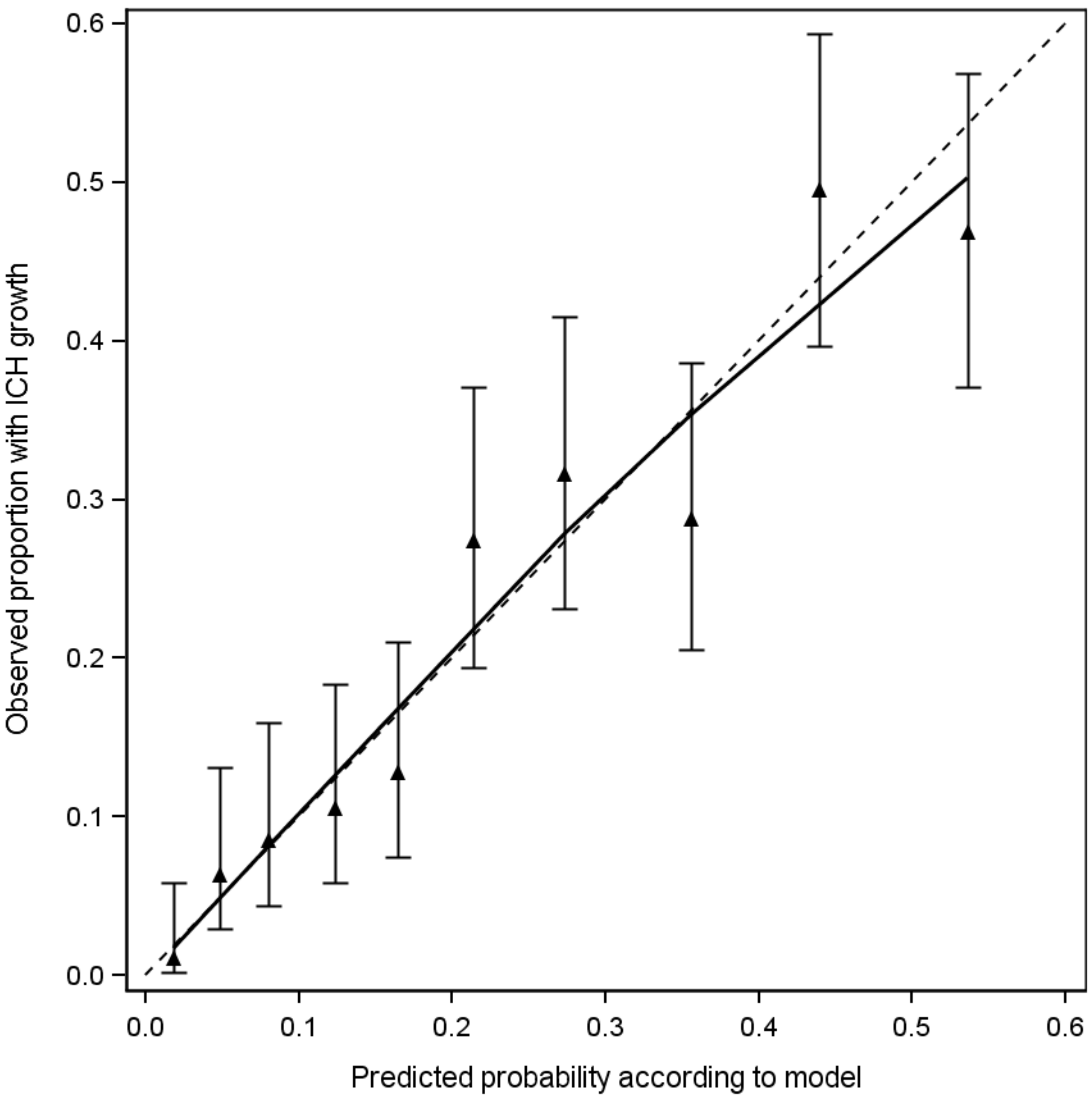

Calibration of the prediction model (derived patients who were not taking anticoagulant therapy at ICH onset) in patients who were taking anticoagulant therapy at ICH onset

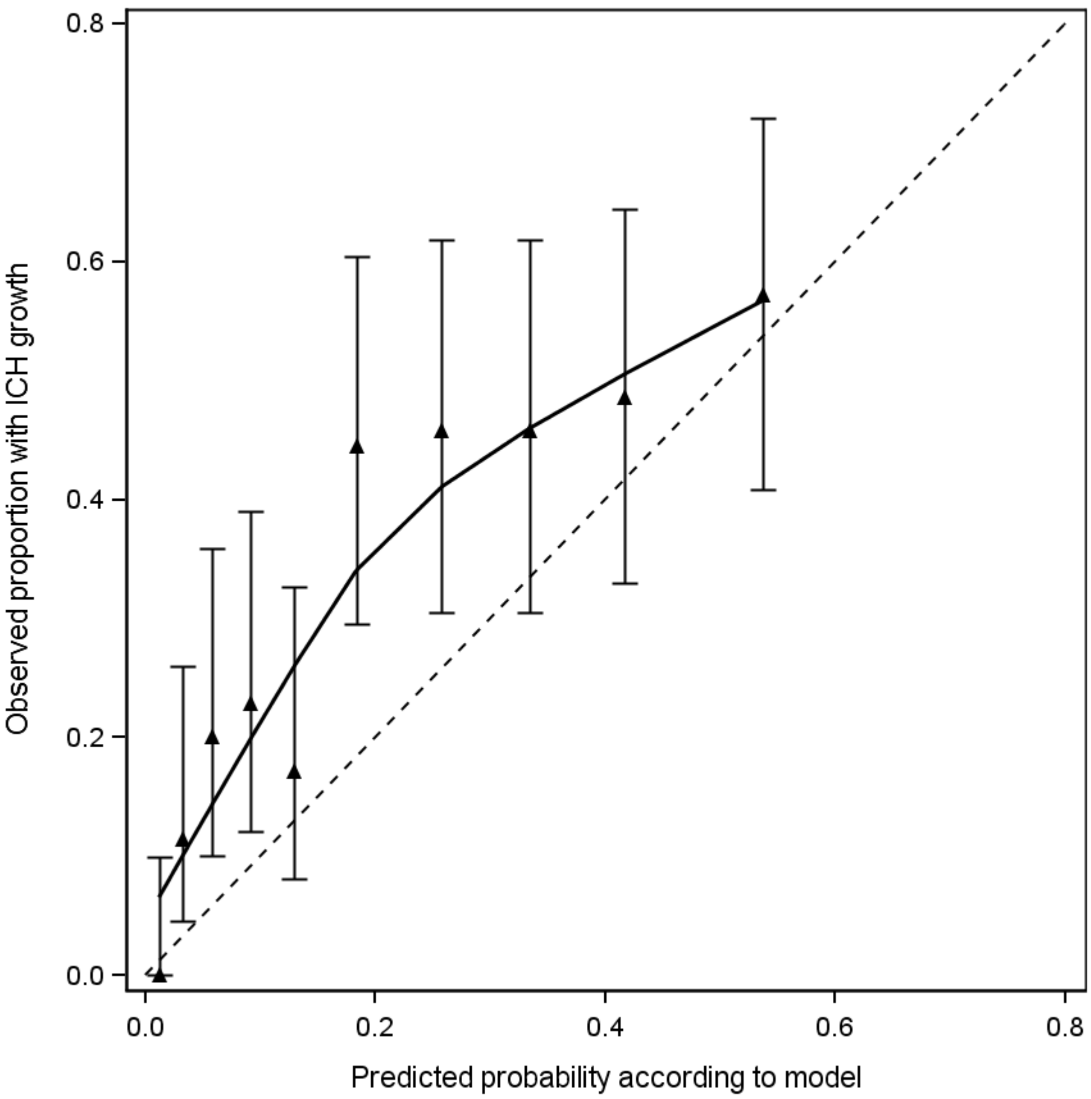

Characteristics of patients whose use of anticoagulant therapy at ICH onset was known, who were included in the development and validation of the second model predicting ICH growth >6ml

Characteristic	Category or values	Development (n = 2,381)	Validation (n = 895)
Sex	Total	2,381	895
	Male	1,422 (60%)	519 (58%)
Age (years)	Total	2,381	895
	Median (lower & upper quartile)	69 (58, 78)	68 (55, 79)
Previous stroke	Total	2,377	873
	Yes	356 (15%)	122 (14%)
Previous ICH	Total	1,120	873
	Yes	62 (6%)	50 (6%)
Previous ischaemic stroke	Total	1,120	873
	Yes	132 (12%)	79 (9%)
History of hypertension	Total	2,380	893
	Yes	1,868 (78%)	632 (71%)
History of diabetes mellitus	Total	2,378	873
	Yes	415 (17%)	154 (18%)
History of liver disease	Total	1,458	464
	Yes	74 (5%)	22 (5%)
History of excessive alcohol consumption	Total	373	861
	Yes	92 (25%)	101 (12%)
Anticoagulant therapy at symptom onset	Total	2,381	895
	Yes	192 (8%)	122 (14%)
Antiplatelet therapy at symptom onset	Total	2,381	895
	Yes	535 (22%)	253 (28%)
Systolic blood pressure at presentation (mmHg)	Total	2,368	892
	Median (lower & upper quartile)	177 (160, 196)	173 (150, 200)
Blood glucose at presentation (mmol/l)	Total	2,291	892
	Median (lower & upper quartile)	6·9 (5·9, 8·6)	7·1 (6·0, 9·1)
Platelet count at presentation ($x10^9/l$)	Total	1,357	889
	Median (lower & upper quartile)	223 (188, 268)	219 (178, 268)
Glasgow Coma Scale score at presentation	Total	2,369	872
	3 - 6	121 (5%)	88 (10%)
	7 - 12	530 (22%)	241 (28%)
	13 - 14	613 (26%)	150 (17%)
	15	1,105 (47%)	393 (45%)
NIHSS score at presentation	Total	1,494	250
	Median (lower & upper quartile)	12 (8, 17)	13 (6, 18)
Time from symptom onset to baseline scan (hours)	Total	2,381	895
	Median (lower & upper quartile)	2·0 (1·2, 3·5)	2·6 (1·4, 5·2)
ICH volume on baseline scan (ml)	Total	2,381	895
	Median (lower & upper quartile)	13 (7, 29)	14 (6, 35)
Location of ICH on baseline scan	Total	2,273	892
	Lobar	496 (22%)	303 (34%)
IVH present on baseline scan	Total	2,283	895
	Yes	790 (35%)	350 (39%)
ICH growth >6ml	Total	2,381	895
	Yes	526 (22%)	209 (23%)

Calibration of the second prediction model in the validation dataset of patients whose use of anticoagulant therapy at ICH onset was known

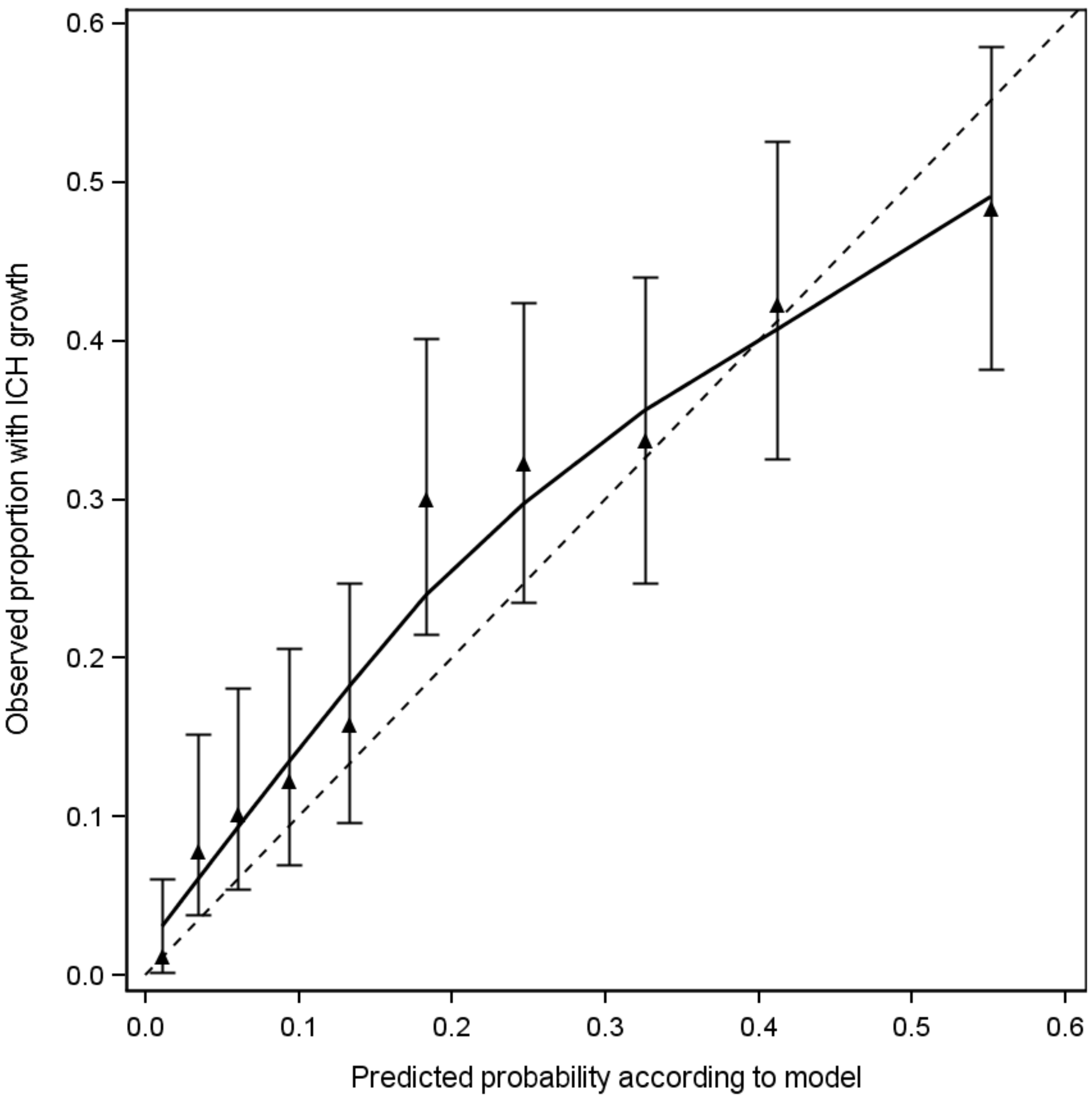

Characteristics of patients who underwent CT angiography to assess the presence of the spot sign, with data available to assess performance of a multivariable model also including use of anticoagulant therapy at ICH onset

**Characteristic**	**Category or values**	**Prediction (n = 837)**
Sex	Total	837
	Male	470 (56%)
Age (years)	Total	837
	Median (lower & upper quartile)	70 (57, 79)
Previous stroke	Total	804
	Yes	94 (12%)
Previous ICH	Total	804
	Yes	29 (4%)
Previous ischaemic stroke	Total	804
	Yes	70 (9%)
History of hypertension	Total	835
	Yes	601 (72%)
History of diabetes mellitus	Total	804
	Yes	137 (17%)
History of liver disease	Total	333
	Yes	7 (2%)
History of excessive alcohol consumption	Total	531
	Yes	69 (13%)
Anticoagulant therapy at symptom onset	Total	837
	Yes	87 (10%)
Antiplatelet therapy at symptom onset	Total	837
	Yes	225 (27%)
Systolic blood pressure at presentation (mmHg)	Total	829
	Median (lower & upper quartile)	174 (150, 198)
Blood glucose at presentation (mmol/l)	Total	833
	Median (lower & upper quartile)	7·2 (6·1, 8·9)
Platelet count at presentation ($x10^9/l$)	Total	831
	Median (lower & upper quartile)	228 (182, 275)
Glasgow Coma Scale score at presentation	Total	803
	3 - 6	62 (8%)
	7 - 12	182 (23%)
	13 - 14	148 (18%)
	15	411 (51%)
NIHSS score at presentation	Total	322
	Median (lower & upper quartile)	14 (7, 18)
Time from symptom onset to baseline scan (hours)	Total	837
	Median (lower & upper quartile)	2·9 (1·5, 5·1)
ICH volume on baseline scan (ml)	Total	837
	Median (lower & upper quartile)	15 (6, 33)
Location of ICH on baseline scan	Total	835
	Lobar	259 (31%)
IVH present on baseline scan	Total	837
	Yes	330 (39%)
CTA spot sign	Total	837
	Present	197 (24%)
ICH growth >6ml	Total	837
	Yes	172 (21%)

Performance of a model to predict ICH growth >6ml including time from symptom onset to baseline imaging, ICH volume on baseline imaging, antiplatelet therapy at symptom onset, and anticoagulant therapy at symptom onset with or without CT angiography

Model	Cut point for predicted probability of ICH growth	Sensitivity	Specificity	Positive predictive value	Negative predictive value
Time from symptom onset to baseline imaging, ICH volume on baseline imaging, antiplatelet therapy at symptom onset, and anticoagulant therapy at symptom onset	0·1	93·6 (88·9 to 96·4)	45·1 (41·4 to 48·9)	30·6 (26·8 to 34·7)	96·5 (93·8 to 98·0)
	0·2	76·2 (69·3 to 81·9)	66·0 (62·3 to 69·5)	36·7 (31·9 to 41·8)	91·5 (88·6 to 93·6)
	0·3	57·0 (49·5 to 64·1)	80·9 (77·7 to 83·7)	43·6 (37·2 to 50·1)	87·9 (85·1 to 90·3)
	0·4	31·4 (24·9 to 38·7)	90·1 (87·6 to 92·1)	45·0 (36·4 to 53·9)	83·5 (80·7 to 86·1)
	0·5	14·0 (9·6 to 19·9)	95·8 (94·0 to 97·1)	46·2 (33·3 to 59·5)	81·2 (78·3 to 83·7)
Time from symptom onset to baseline imaging, ICH volume on baseline imaging, antiplatelet therapy at symptom onset, anticoagulant therapy at symptom onset, and assessment of spot sign on CT angiography	0·1	92·4 (87·5 to 95·5)	53·7 (49·9 to 57·4)	34·1 (29·9 to 38·5)	96·5 (94·1 to 97·9)
	0·2	80·2 (73·6 to 85·5)	74·3 (70·8 to 77·5)	44·7 (39·2 to 50·2)	93·6 (91·1 to 95·4)
	0·3	57·0 (49·5 to 64·1)	85·7 (82·8 to 88·2)	50·8 (43·8 to 57·7)	88·5 (85·8 to 90·7)
	0·4	47·1 (39·8 to 54·5)	88·9 (86·3 to 91·0)	52·3 (44·4 to 60·0)	86·7 (83·9 to 89·0)
	0·5	35·5 (28·7 to 42·9)	91·9 (89·6 to 93·7)	53·0 (44·0 to 61·9)	84·6 (81·8 to 87·1)

Associations between each predictor and ICH growth >6ml or relative increase >33% in patients who were not taking anticoagulant therapy at symptom onset. The adjusted odds ratio for each predictor is adjusted for both time from symptom onset to baseline scan and ICH volume on baseline scan.

			Unadjusted		Adjusted	
**Predictor**	**Number of patients (cohorts)**	**Comparison**	**Odds ratio (95% CI)**	**p-value**	**Odds ratio (95% CI)**	**p-value**
Sex	4,884 (35)	Male versus Female	1·16 (1·01 to 1·32)	0·035	1·18 (1·02 to 1·35)	0·021
Age (years)	5,074 (36)	76 versus 56	1·04 (0·94 to 1·15)	0·44	1·06 (0·96 to 1·18)	0·26
Previous stroke	4,471 (29)	Yes versus No	1·14 (0·94 to 1·39)	0·19	1·22 (0·99 to 1·50)	0·060
Previous ICH	2,542 (16)	Yes versus No	1·12 (0·79 to 1·59)	0·54	1·09 (0·75 to 1·57)	0·66
Previous ischaemic stroke	2,666 (21)	Yes versus No	1·27 (0·94 to 1·71)	0·11	1·36 (1·00 to 1·86)	0·053
History of hypertension	5,050 (36)	Yes versus No	0·88 (0·75 to 1·02)	0·082	0·91 (0·78 to 1·07)	0·26
History of diabetes mellitus	4,197 (31)	Yes versus No	1·07 (0·89 to 1·28)	0·49	1·07 (0·89 to 1·30)	0·46
History of liver disease	3,248 (19)	Yes versus No	1·80 (1·34 to 2·40)	<0·0001	1·80 (1·34 to 2·43)	0·0001
History of excessive alcohol consumption	3,029 (23)	Yes versus No	1·24 (1·00 to 1·54)	0·050	1·21 (0·97 to 1·52)	0·090
Antiplatelet therapy at symptom onset	4,170 (31)	Yes versus No	1·34 (1·13 to 1·60)	0·0009	1·47 (1·23 to 1·76)	<0·0001
Systolic blood pressure at presentation (mmHg)	4,882 (36)	198 versus 158	1·05 (0·97 to 1·15)	0·22	1·02 (0·93 to 1·12)	0·64
Blood glucose at presentation (mmol/l)	4,265 (36)	8·7 versus 5·9	1·08 (1·01 to 1·15)	0·021	1·01 (0·95 to 1·09)	0·70
Platelet count at presentation (x10^9/l)	3,857 (36)	266 versus 181	0·84 (0·79 to 0·89)	<0·0001	0·83 (0·78 to 0·89)	<0·0001
Glasgow Coma Scale score at presentation	4,564 (32)	3 - 6 versus 15	2·25 (1·65 to 3·07)	<0·0001	1·40 (0·99 to 1·98)	0·086
		7 - 12 versus 15	1·86 (1·54 to 2·26)		1·29 (1·03 to 1·62)	
		13 - 14 versus 15	1·42 (1·17 to 1·73)		1·19 (0·96 to 1·48)	
NIHSS score at presentation	2,661 (35)	18 versus 7	1·98 (1·72 to 2·27)	<0·0001	1·56 (1·33 to 1·82)	<0·0001
Location of ICH on baseline scan	4,920 (35)	Lobar versus Non-lobar	1·31 (1·12 to 1·54)	0·0008	1·11 (0·93 to 1·33)	0·23
IVH present on baseline scan	4,980 (36)	Yes versus No	1·01 (0·88 to 1·17)	0·85	0·86 (0·74 to 1·00)	0·053

Receiver operating characteristic (ROC) curves for the predicted probability of ICH growth >6ml or relative increase >33% using four predictors (solid line) and four predictors plus CT angiography spot sign (dashed line)

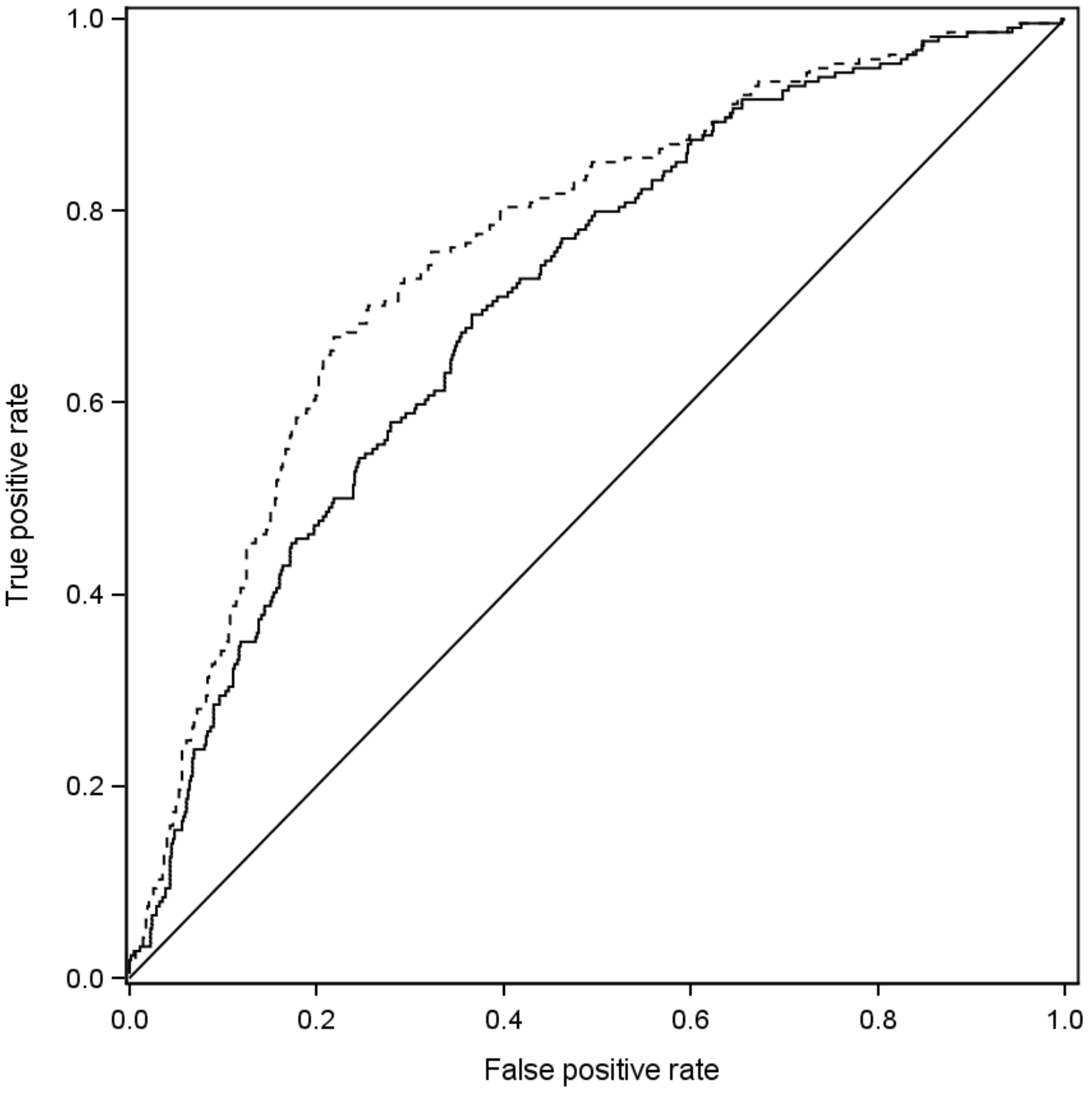

Predicted probability of ICH growth >6ml according to time from symptom onset to baseline imaging in patients who were not taking anticoagulant therapy at symptom onset by volumetric method used to calculate ICH volumes (2,547 patients from 15 cohorts which used planimetric methods versus 2,529 patients from 21 cohorts which used ABC/2 or mostly ABC/2 method)

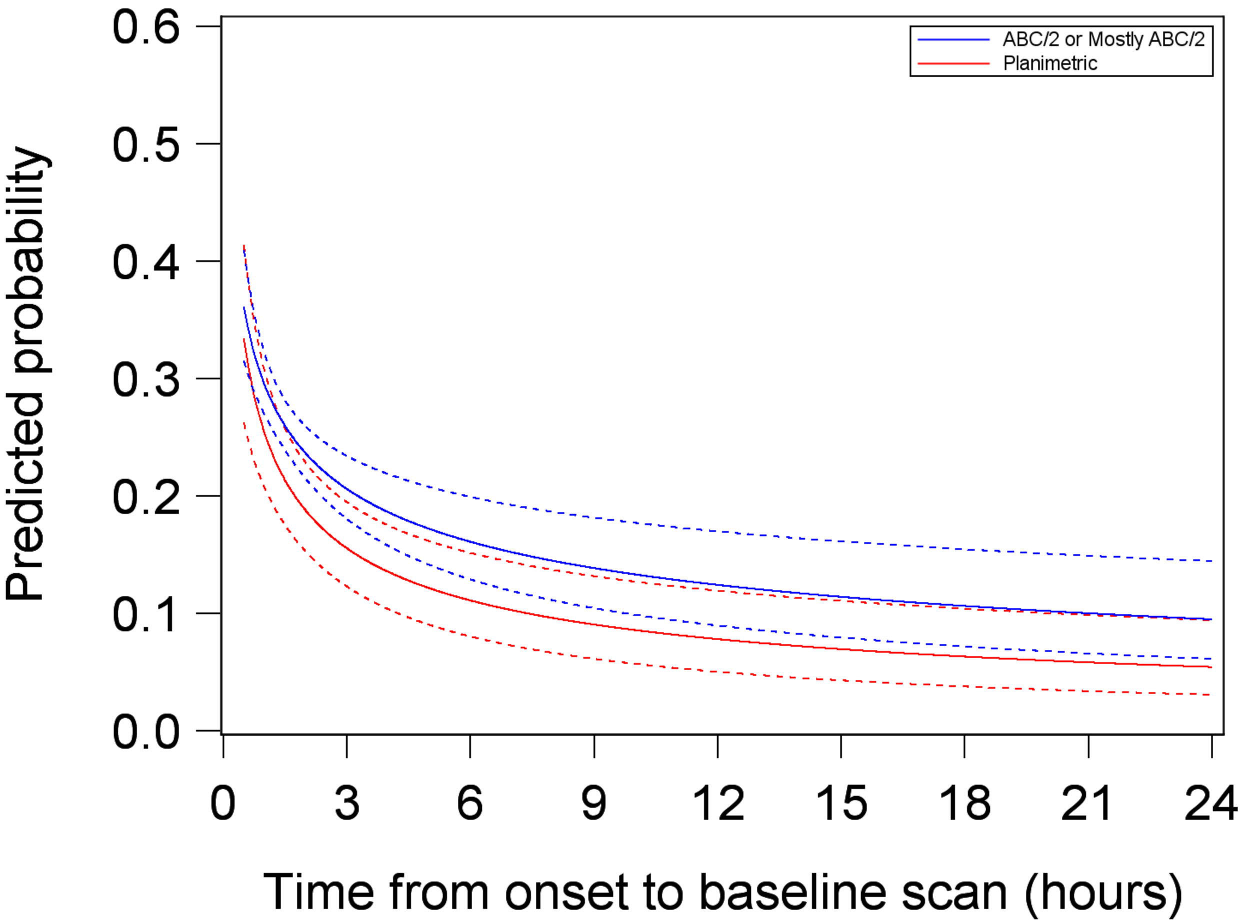

Although the predicted probability of ICH growth was slightly higher in the subgroup of patients from cohorts which used the ABC/2 or mostly ABC/2 method compared to the subgroup of patients from cohorts which used the planimetric method to calculate ICH volumes, there is no evidence that the risk of ICH growth differs with volumetric method and/or the shape of the relationship between time from onset to baseline scan and ICH growth is modified by the volumetric method used. The best fitting fractional polynomial for modelling the relationship between time from onset to baseline scan and ICH growth is identical in the two subgroups of patients and when terms for volumetric method ($p=0.088$), interaction between volumetric method and time from onset to baseline scan ($p=0.13$), or both ($p=0.16$) are added to the logistic regression model for ICH growth none of these give a statistically significant improvement to the model.

Predicted probability of ICH growth >6ml according to ICH volume on baseline imaging in patients who were not taking anticoagulant therapy at symptom onset by volumetric method used to calculate ICH volumes (2,547 patients from 15 cohorts which used planimetric methods versus 2,529 patients from 21 cohorts which used ABC/2 or mostly ABC/2 method)

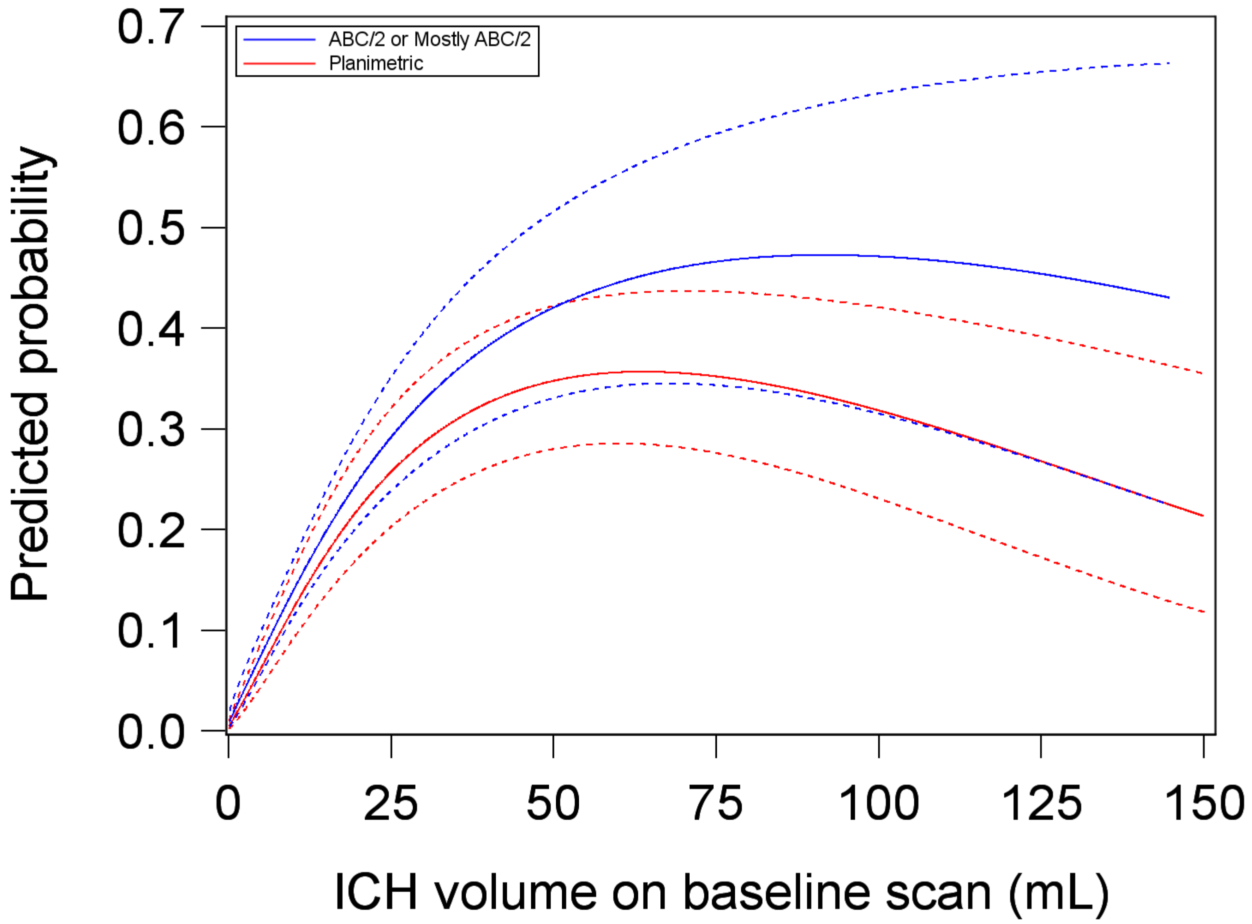

Although the predicted probability of ICH growth was higher in the subgroup of patients from cohorts which used the ABC/2 or mostly ABC/2 method compared to the subgroup of patients from cohorts which used the planimetric method to calculate ICH volumes, there is no evidence that the risk of ICH growth differs with volumetric method and/or the shape of the relationship between ICH volume on baseline scan and ICH growth is modified by the volumetric method used. The best fitting fractional polynomial for modelling the relationship between ICH volume on baseline scan and ICH growth is identical in the two subgroups of patients and when terms for volumetric method (p=0.42), interaction between volumetric method and ICH volume on baseline scan (p=0.24), or both (p=0.33) are added to the logistic regression model for ICH growth none of these give a statistically significant improvement to the model.

Predicted probability of ICH growth >6ml according to time from symptom onset to baseline imaging in patients who were not taking anticoagulant therapy at symptom onset by cohort epoch (2,429 patients from 18 cohorts in the first epoch [1994-2007] versus 2,647 patients from 18 cohorts in the second epoch [2008-2015])

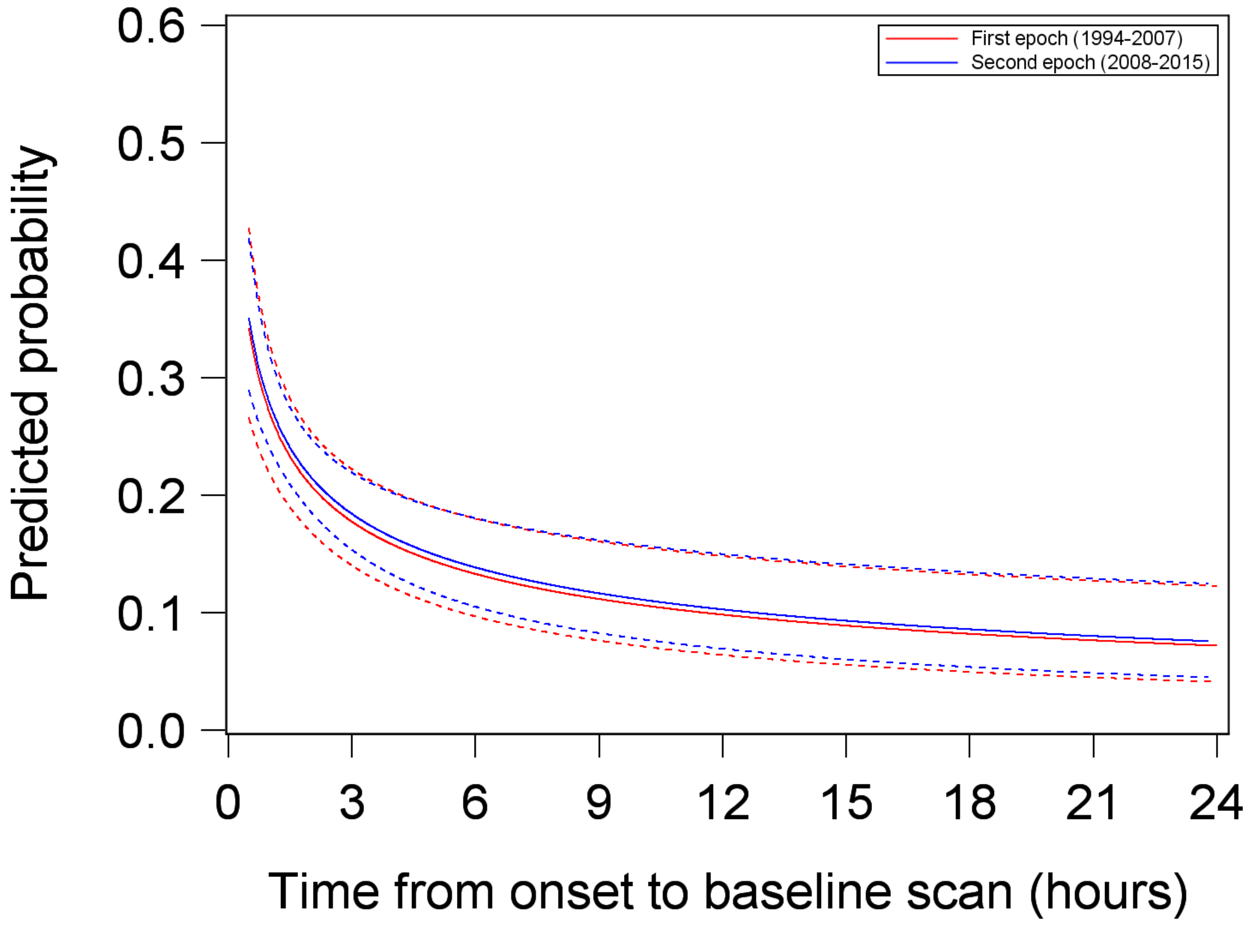

The predicted probability of ICH growth was very similar in the subgroups of patients from cohorts in the first epoch (1994-2007) and the second epoch (2008-2015). There is no evidence that the risk of ICH growth differs with epoch and/or the shape of the relationship between time from onset to baseline scan and ICH growth is modified by the epoch based on end date of recruitment to each cohort. The best fitting fractional polynomial for modelling the relationship between time from onset to baseline scan and ICH growth is identical in the two subgroups of patients and when terms for epoch (p=0.75), interaction between epoch and time from onset to baseline scan (p=0.86), or both (p=0.95) are added to the logistic regression model for ICH growth none of these give a statistically significant improvement to the model.

Predicted probability of ICH growth >6ml according to ICH volume on baseline imaging in patients who were not taking anticoagulant therapy at symptom onset by cohort epoch (2,429 patients from 18 cohorts in the first epoch [1994-2007] versus 2,647 patients from 18 cohorts in the second epoch [2008-2015])

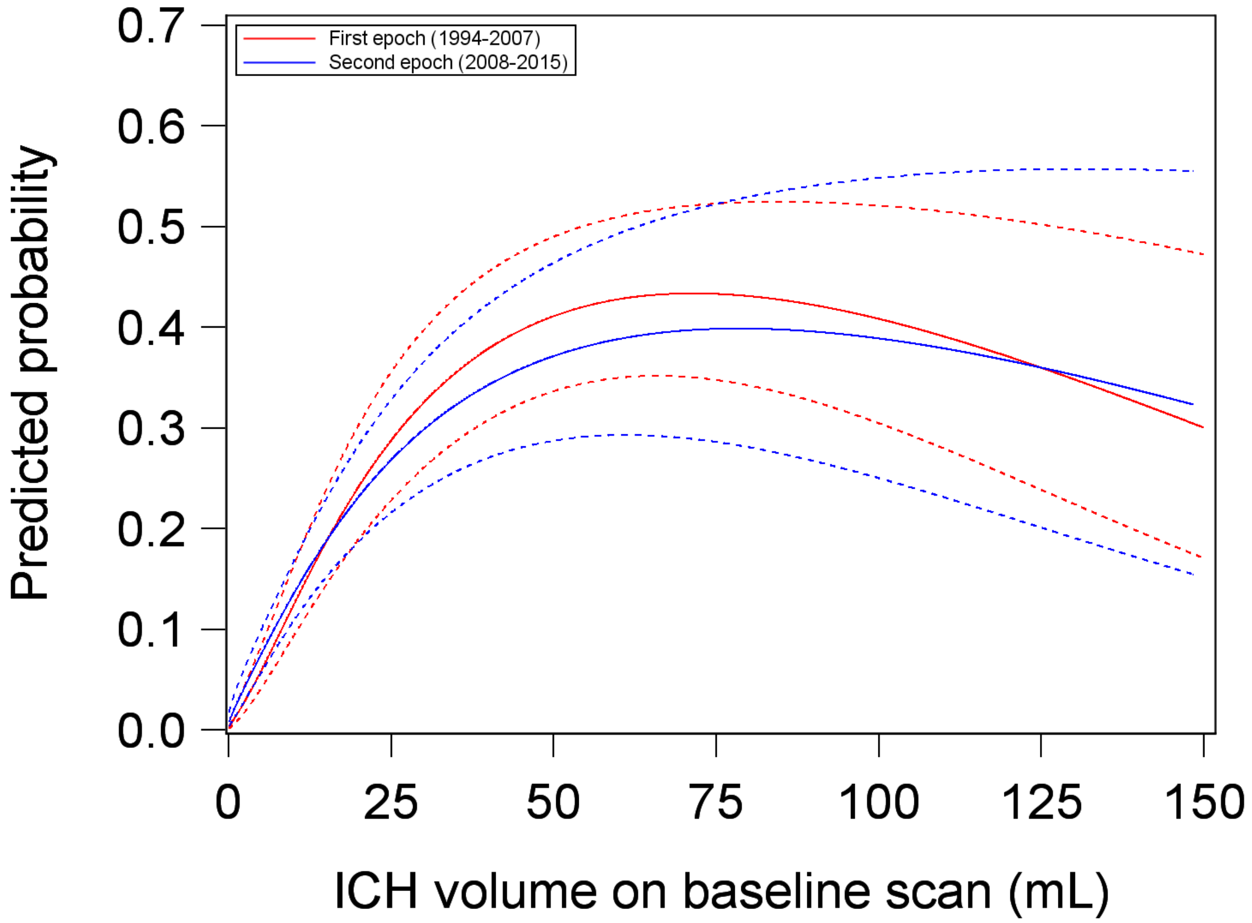

The predicted probability of ICH growth was reasonably similar in the subgroups of patients from cohorts in the first epoch (1994-2007) and the second epoch (2008-2015). There is no evidence that the risk of ICH growth differs with epoch and/or the shape of the relationship between ICH volume on baseline scan and ICH growth is modified by the epoch based on end date of recruitment to each cohort. The best fitting fractional polynomial for modelling the relationship between ICH volume on baseline scan and ICH growth is identical in the two subgroups of patients and when terms for epoch (p=0.86), interaction between epoch and time from onset to baseline scan (p=0.65), or both (p=0.42) are added to the logistic regression model for ICH growth none of these give a statistically significant improvement to the model.

Collaborators

VISTA-ICH Collaborators: Dan F. Hanley Jr (Chair), Kenneth S. Butcher, Stephen Davis, Barbara Gregson, Kennedy R. Lees, Patrick D. Lyden, Stephan A. Mayer, Keith W. Muir, and Thorsten Steiner.

ICH Growth Individual Patient Data Meta-analysis Collaborators: Peng Xie, Babak Bakhshayesh, Mark McDonald, Thomas Brott, Paolo Pennati, Adrian R. Parry-Jones, Craig J. Smith, Stephen J. Hopkins, Mark Slevin, Veronica Campi, Puneetpal Singh, Francesca Papa, Aurel Popa-Wagner, Valeria Tudorica, Ryo Takagi, Akira Teramoto, Karin Weissenborn and Heinrich Lanfermann.

Affiliations: Brain Injury Outcomes, Johns Hopkins University, USA (Prof DF Hanley Jr MD); WMC Health Sciences Center, Edmonton, Alberta, Canada (Prof KS Butcher PhD); Melbourne Brain Centre, Royal Melbourne Hospital and University of Melbourne, Australia (Prof S Davis MD); Neurosurgical Trials Group, Institute of Neuroscience, Newcastle University, Newcastle upon Tyne, UK (B Gregson PhD); Institute of Cardiovascular & Medical Sciences, University of Glasgow, UK (Prof KR Lees FRCP), Cedars-Sinai Medical Centre, Los Angeles, California, USA (Prof PD Lyden MD), Department of Neurology, Neuroscience Institute, Henry Ford Health System, Detroit, USA (Prof SA Mayer MD), Institute of Neuroscience & Psychology, University of Glasgow, Queen Elizabeth University Hospital, Glasgow, UK (Prof KW Muir MD); Department of Neurology, Klinikum Frankfurt Höchst, Frankfurt, Germany (Prof T Steiner MD); Department of Neurology, The First Affiliated Hospital of Chongqing Medical University, Chongqing, China (P Xie MD); Department of Neurology, Poursina Hospital, Guilan University of Medical Sciences, Rasht, Iran (B Bakhshayesh MD); Department of Neurology, McGovern Medical School at the University of Texas Health Science Center, Houston, TX, USA (M McDonald BS); University of Cincinnati (Ohio) Medical Centre, Ohio, USA (T Brott MD); Emergency Department, Cecina Hospital, Cecina, Italy (P Pennati, MD); Vascular and Stroke Centre, Manchester Academic Health Science Centre, Salford Royal NHS Foundation Trust, Salford, United Kingdom (AR Parry-Jones, PhD; CJ Smith, MD; SJ Hopkins, PhD); SBCHS, Manchester Metropolitan University, Manchester, United Kingdom and Institut Catal. de Ci.ncies Cardiovasculars Hospital de la Santa Creu i Sant Pau, Pavell. del Convent Sant Antoni Maria Claret, Barcelona, Spain (M Slevin, PhD); Central Laboratory, Sanatorio Pasteur, Catamarca, Argentina (V Campi, PhD); Molecular Genetics Laboratory, Department of Human Genetics, Punjabi University, Patiala, India (P Singh, PhD); Neurological Section, SMDN—Center for Cardiovascular Medicine and Cerebrovascular Disease Prevention, Sulmona, L'Aquila, Italy (F Papa, MD); Research Center for Microscopic Morphology and Immunology & Chair of Biochemistry, University of Medicine and Pharmacy of Craiova, Craiova, Romania and Molecular Psychiatry, Department of Psychiatry, Medical University Rostock, Rostock, Germany (A Popa-Wagner, PhD); Department of Neurology, University of Medicine and Pharmacy, Craiova, Romania (V Tudorica); Department of Radiology, Nippon Medical School, Tokyo, Japan (R Takagi, MD); Department of Neurological Surgery, Nippon Medical School, Tokyo, Japan (A Teramoto, MD); Department of Neurology, Hannover Medical School, Carl-Neuberg-Str. 1, 30625 Hannover, Germany (K Weissenborn, MD); Institute of Diagnostic and Interventional Neuroradiology, Hannover Medical School, Carl-Neuberg.Str. 1, 30625 Hannover, Germany (H Lanfermann, MD).
